# Exergy and Exergoeconomic Analysis of the Gas Subcooled Process for the Brazilian Market: An Approach to Enhance Natural Gas Liquids Recovery

**DOI:** 10.1021/acsomega.5c02484

**Published:** 2025-06-24

**Authors:** Isidro Alejandro Argueta Flores, Ana Paula Meneguelo, Cintia Marangoni, Yuri Nascimento Nariyoshi, Marcelo Silveira Bacelos

**Affiliations:** † Departamento de Engenharias e Tecnologias, Programa de Pós-graduação em Energia, 28126 Universidade Federal do Espírito Santo, Rod. BR 101 Norte, Km. 60, 29932-540 São Mateus, Espírito Santo, Brazil; ‡ Departamento de Engenharia Química e Alimentos, Programa de Pós-graduação em Engenharia Química e Alimentos, Campus Universitário, 28117 Universidade Federal de Santa Catarina, Trindade, 88040-900 Florianópolis, Santa Catarina, Brazils; § Departamento de Engenharia Ambiental, Campus Universitário, Universidade Federal do Espírito Santo, Av. Fernando Ferrari 514, 29075-910 Vitória, Espírito Santo, Brazil

## Abstract

The gas subcooled process (GSP) is the most widely adopted natural gas liquid (NGL) recovery method due to its adaptability to varying gas compositions and its alignment with the market emphasis in Brazil on lighter hydrocarbons. However, the process remains limited by high energy consumption and significant thermodynamic inefficiencies, particularly in the expansion and separation stages. To address these drawbacks, this research employs a detailed exergy and exergoeconomic assessment of four GSP configurations, termed Turbo-Expander (TE), Joule–Thomson (JT), Turbo-Expander with Mechanical Refrigeration (TEMR), and Joule-Thomson with Mechanical Refrigeration (JTMR), to identify and quantify the sources of energy degradation and their associated economic impacts. The results reveal that the TE configuration achieves the highest exergy efficiency (71%) and demonstrates superior cost performance by maximizing the shaft work recovery. Conversely, the TEMR setup incurs the highest exergy destruction cost (US $ 5687/h), primarily due to its energy-intensive refrigeration cycle. By integrating thermodynamic and economic diagnostics at the equipment level, these research findings provide actionable insights into improving GSP system design, support infrastructure modernization, and guide investment decisions, thereby contributing directly to the advancement of a more sustainable and competitive natural gas sector in Brazil.

## Graphical abstract

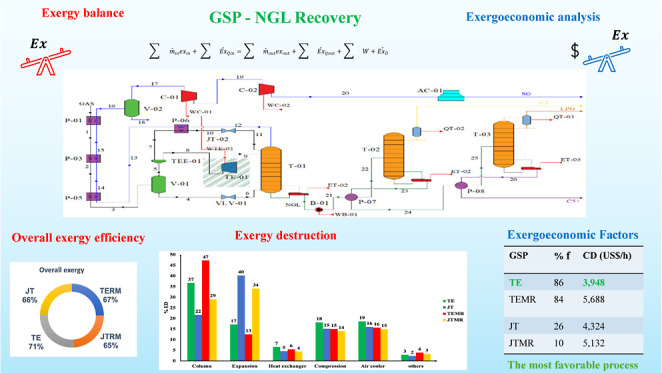

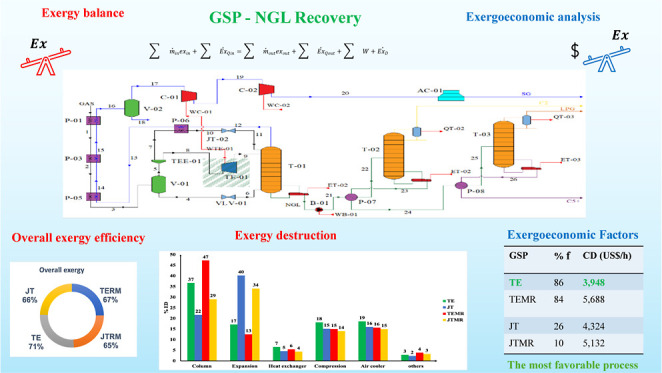

## 1 Introduction

Transitioning to cleaner and renewable energy sources is essential for reducing CO_2_ emissions. However, projections from the International Energy Agency (IEA) indicate that emerging renewable technologies, such as hydrogen, solar, and wind, will not be sufficient to fully meet global energy demand by 2050.^1^ As a result, natural gas (NG) is expected to play a crucial role in the coming decades, given that its CO_2_ emissions are approximately 45% lower than oil.^2^

In Brazil, NG currently accounts for 10.5% of the national energy matrix and 8.4% of electricity generation.^3^ The country still has the potential to quadruple its NG production by 2050 compared to current levels.^4^ The Brazilian projected production, coupled with infrastructure constraints, underscores the need for process analysis and development. Key focus areas include gas treatment, natural gas liquids (NGL) recovery, and gas liquefaction or compression.^5^ Nevertheless, critical challenges persist, including underdeveloped infrastructure, limited competitiveness, regulatory gaps, and high dependence on Petrobras, which controls over 90% of the NG processing capacity. These structural issues, particularly the lack of Natural Gas Processing Plants (NGPPs) for presalt gas conditioning, contribute to the reinjection of large volumes of associated gas.^6,7^ Moreover, the absence of a clear pricing tariff model and performance standards for Natural Gas Liquids (NGLs) recovery technologies deters private investment and limits modernization efforts.

Cryogenic NGL recovery, based on the industry-standard single-stage separation (ISS) process, removes methane to produce NGL, which can be sold or further fractionated.^8^ Despite the cryogenic high purity and recovery rates compared to other methods, they are energy-intensive. Only a few are licensed under U.S. Patents, with the most prominent ones developed by the Ortloff and IPSI companies. Ortloff holds Patents for the Gas Subcooled Process (GSP), Cold Residue Recycle (CRR), and Recycle Vapor-Split (RSV) methods, while IPSI Company owns the enhanced recovery processes IPSI-1 and IPSI-2, which are widely recognized for their industrial application.^9^ The GSP, commonly used for ethane recovery, provides a more flexible and robust alternative. It minimizes losses of propane and heavier hydrocarbons, while its modular design allows for the processing of varying gas compositions under different operating conditions.^10^ Alternative ethane recovery technologies include the Cold Residue Reflux (CRR) and Recycle Split Vapor (RSV) processes, offering high recovery rates while involving complex equipment configurations and increased energy consumption.^11,12^ The IPSI-1 and IPSI-2 processes provide high recovery efficiency for heavier hydrocarbons.^13^ These processes are advanced cryogenic methods that improve NGL recovery by enhancing operations at the bottom of the demethanizer column. IPSI-1 uses a self-refrigeration system to minimize external cooling requirements and enhance separation efficiency, while IPSI-2 incorporates a closed-loop cycle for improved thermal integration.

Although IPSI-1 and IPSI-2 present these advantages, they become relevant for offshore and space-constrained applications. The decentralized production of Brazil, with a market focus on methane, ethane, and propane rather than heavier components, reduces the economic feasibility of implementing IPSI systems.^14^

The literature highlights the process optimization in LPG and NGL production, demonstrating that selecting appropriate refrigeration and expansion configurations, coupled with advanced optimization techniques, can enhance the exergy efficiency while reducing specific energy consumption.

Mazumder et al.^15^ developed a novel dual mixed refrigerant (DMR) LNG liquefaction process, integrating turbo-expansion and Joule–Thompson expansion technologies. The authors demonstrated that using ethane and propane as the mixed refrigerant, along with energy recovery through a Turbo-Expander, enhances the efficiency. Compared with the conventional propane-precooled mixed refrigerant process, the proposed design reduces capital costs by 12.9% and operating costs by 7.4%, highlighting its economic and thermodynamic advantages.

Almeida-Trasvina et al.^16^ and Almeida-Trasvina and Smith^17^ investigated the optimization of single mixed refrigerant (SMR) cycles for small-scale liquefied natural gas (LNG) production, focusing on minimizing shaft power demand in refrigerant compression, a major contributor to operating costs. Both studies analyzed structural modifications to SMR cycles and employed optimization techniques to enhance the energy efficiency. The findings demonstrated that modifying the cycle configuration and optimizing the refrigerant composition could significantly reduce the shaft work demand. In particular, one study first showed that a novel SMR cycle achieved a 10% reduction in shaft work demand compared to the PRICO cycle; while the other, in sequence, provided a thermodynamic-based optimization approach to improve design methodologies and enhance energy efficiency across different SMR configurations.

Kim and Gundersen^18^ investigated three process configurations for LPG and NGL production, using exergy efficiency and specific energy consumption as objective functions for optimization. The authors indicate that the first configuration, which combines a dual mixed refrigerant (DMR) cycle for LPG and an internal subcooling system (ISS) for NGL, demonstrated the best performance. This configuration exhibited the lowest specific energy consumption (214.98 to 216.55 kW h/ton) and the highest exergy efficiency (68.97–69.50%). They concluded that reducing specific energy consumption leads to a higher exergy efficiency, making this configuration particularly promising for GSP and RSV technologies used in NGL extraction.

Khajehpour et al.^19^ optimized an NGL recovery plant by comparing three refrigeration system expansion methods: Joule–Thompson, Turbo-Expander, and hybrid Joule–Thompson and Turbo-Expander configuration. They identified the Joule–Thompson expansion as the most efficient when considering the natural gas feed temperature. However, all expansion systems exhibited low exergy efficiency within the 30–40 °C feed temperature range. The researchers applied Sequential Quadratic Programming (SQP) and a genetic algorithm, which resulted in 12% and 15% improvement in exergy efficiency, respectively. The authors pointed out that optimizing compressor pressure differentials and maximizing pressure differences in expanders can reduce exergy destruction and enhance the overall efficiency.

He and Lin^20^ designed and optimized a liquefied natural gas (LNG) process flow diagram for high-ethane-content gas. Their proposed process utilized two mixed refrigerant systems—one for precooling and another for liquefaction. Three scenarios were assessed: the original process, a modified liquefaction refrigeration system, and an additional compression stage. The optimization aimed to minimize energy consumption, with results showing that the third scenario had the lowest energy consumption. Furthermore, the authors observed that as the ethane content increased, the specific energy consumption decreased linearly, while exergy efficiency remained stable across varying ethane concentrations and refrigerant compositions.

Among cryogenic methods, the GSP is the most widely deployed in Brazil, owing to its flexibility in handling variable gas compositions and its compatibility with the Brazilian market focus on methane, ethane, and propane. Conversely, the GSP is energy-intensive and thermodynamically inefficient due to inherent process irreversibilities, requiring analyses on thermodynamic inefficiencies at the equipment level or integrating these losses with cost structures for process improvement.^18–20^ Franco et al.^21^ optimized the energy consumption of a natural gas processing unit—GSP. Various compositions of natural gas were analyzed to determine their impact on energy consumption. The authors employed a minimum recovery rate of 90% for C_3_^+^ as an optimization constraint and observed that manipulation of operational variables can significantly reduce the specific energy consumption. Considering criteria such as energy efficiency, economic viability, recovery rates, production levels, safety, and quality, the authors validated their approach. Nevertheless, the authors did not perform an exergy analysis for individual equipment.

Exergy analysis evaluates the quality and usability of energy within an NGL process, identifying the locations and causes of energy degradation. In addition, exergoeconomic analysis assigns economic values to these thermodynamic inefficiencies, integrating energy quality degradation with capital and operating costs. It enables the identification of key areas for cost reduction and supports informed decision-making aimed at achieving sustainable and efficient process design.^9,10^ For NGL recovery processes, it has shown that separation columns accounted for the highest exergy destruction at 64%, followed by heat exchangers at 15%.^22^ Exergy efficiency can be further correlated with exergoeconomic factors through thermoeconomic analysis.^23^ The exergoeconomic evaluation prioritizes equipment by strategic importance, ranking the Turbo-Expander first, the compressor (noted for high investment cost), and the lasting demethanizer column (also high investment cost). Conversely, the demethanizer column exhibits a relatively low exergy destruction cost.^22^

Recent studies have highlighted the relevance of such an integrated analysis in the NGL process. Shamsi et al.^24^ compared Turbo-Expander (TE), Joule–Thomson (JT), and hybrid refrigeration systems, concluding that TE configurations achieved the highest exergy efficiency (75%) and the lowest carbon footprint. Similarly, Islam et al.^25^ examined six patented NGL processes (ISS, GSP, CRR, RSV, IPSI-1, and IPSI-2) and found that GSP offers an optimal trade-off between capital cost and energy efficiency; however, it has high exergy destruction in demethanizer columns and compression systems.

Despite these advancements, the literature remains limited regarding equipment-level exergoeconomic assessments of the GSP. A recent study conducted by Islam et al.^25^ presents a comparative exergy analysis of six NGL recovery processes, including the GSP. Conversely, it does not include monetary valuation or component-level exergoeconomic diagnostics, which limits its applicability for process cost optimization.

As an innovative approach, this research conducts a detailed exergy and exergoeconomic assessment of four GSP configurations tailored to Brazilian NG conditions: Turbo-Expander (TE), Joule–Thomson (JT), Turbo-Expander with Mechanical Refrigeration (TEMR), and Joule–Thomson with Mechanical Refrigeration (JTMR). Distinct from Junior et al.^26^ and Franco et al.,^21^ this research decomposes the system into individual unit operations (columns, compressors, heat exchangers, and expanders) and evaluates the exergy efficiency and exergoeconomic factor. Furthermore, three distillation columns (demethanizer, de-ethanizer, and debutanizer) are analyzed, transforming the GSP into a flexible unit for producing sales gas (SG), ethane (C_2_), LPG (liquefied petroleum gas), or C_5_^+^ (natural gasoline), expanding upon the analyses carried out by Islam et al.^25^ The analysis identifies the most cost-intensive inefficiencies and provides design insights for achieving optimal trade-offs between thermodynamic performance and economic viability. These insights can inform NG infrastructure development strategies, contributing to a more sustainable and competitive gas market. Natural gas is the fuel for the energy transition. By identifying equipment that shows losses and inefficiencies along with an economic analysis, the research highlights potential efficiency gains that can support the use of more effective sustainability indicators related to natural gas, thereby guiding new investment decisions.

## 2 Methodology

### 2.1 Process Flow Diagram Description

Four arrangements of process flow diagrams (PFDs) were exploited for exergy and exergoeconomic analysis, namely: Turbo-Expander (TE; Figure 1a), Joule–Thompson (JT; Figure 1b), Turbo-Expander with Mechanical Refrigeration (TEMR; Figure 1c), and Joule–Thompson with Mechanical Refrigeration (JTMR; Figure 1d). Figure 1 shows adaptations to the GSP process’s expansion system and refrigeration cycle, which uses a Turbo-Expander before the inlet of the demethanizer column.^27^ Such changes were based on the works of Kherbeck and Chebbi^28^ and Getu et al.^10^ and later adapted to the Brazilian Market by Junior et al.^26^ The PDFs comprise cooling the feed stream with sale gas (in plate heat exchangers) before expanding in the TE or JT valve. This cooling can also be associated with mechanical refrigeration as it occurs in TEMR and JTMR. The sales gas is then separated by distillation from LPG, ethene, and C_5_^+^ and must be stabilized at a pressure of 7.101 kPa and a temperature of 25.00 °C. This conditioning is carried out through compressor C-02 and air cooler AC-01. The TEMR and TE arrangements have an additional compression stage, in which compressor C-01 is driven by the shaft work generated by the Turbo-Expander TE-01.

**Figure 1:**
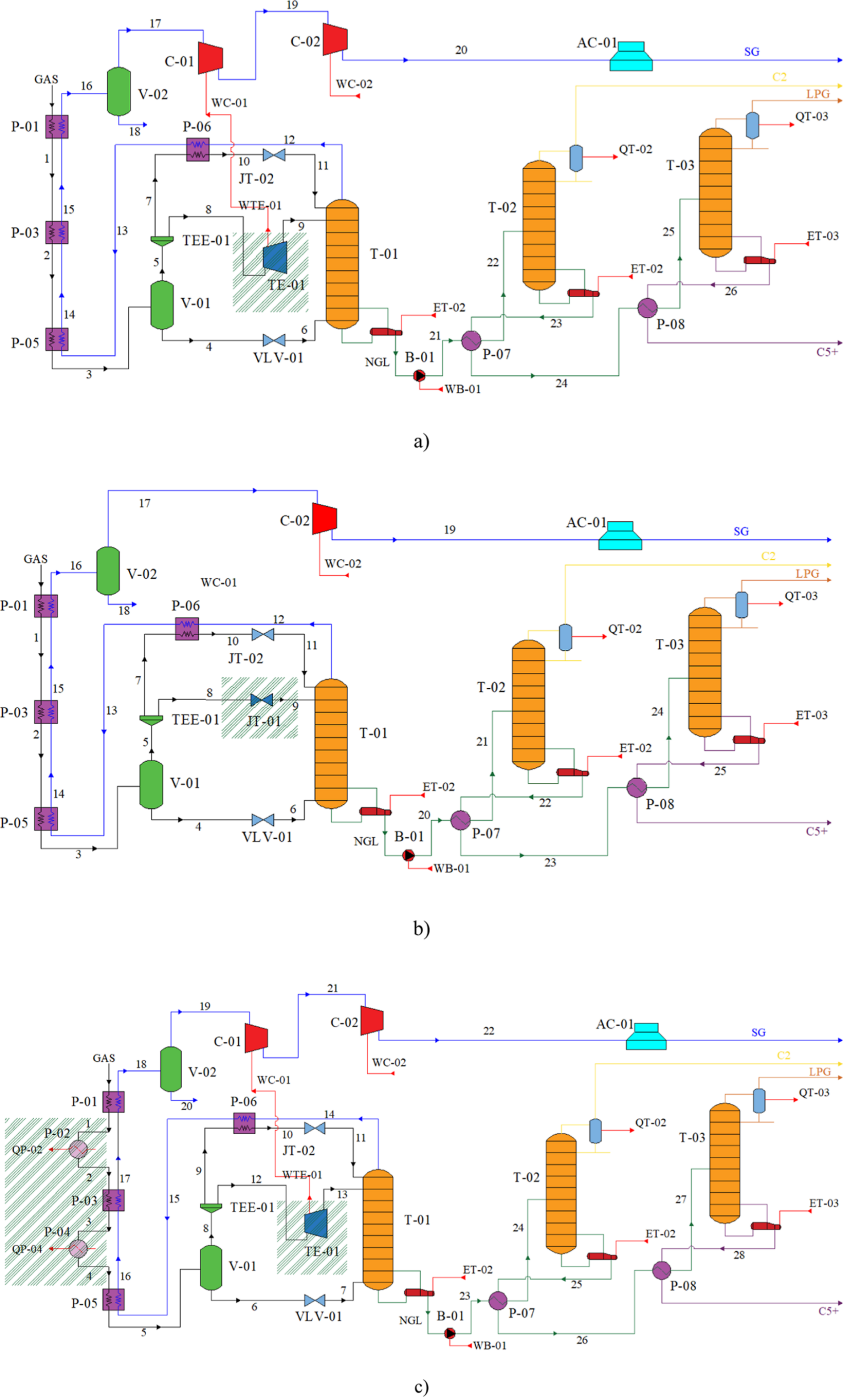

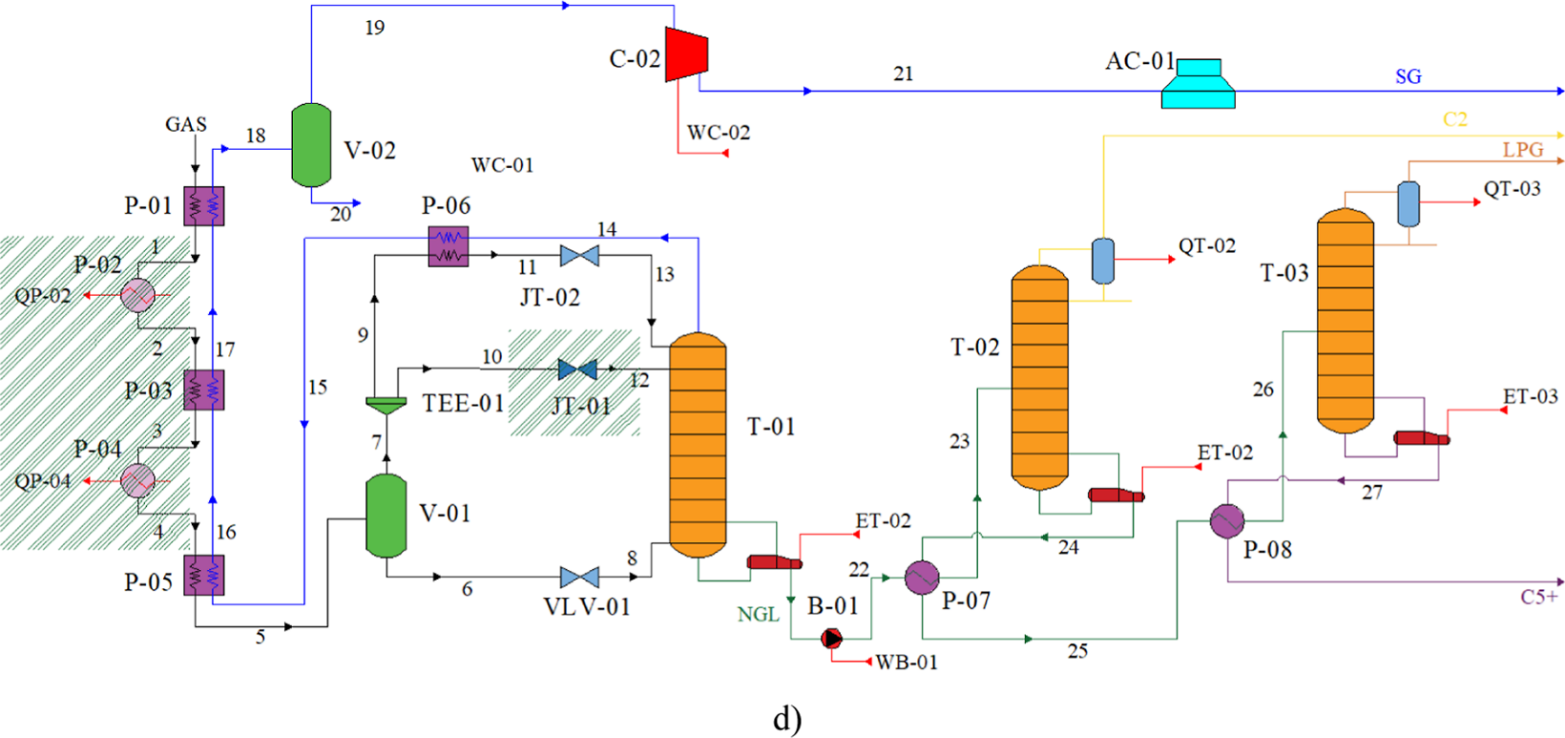
Process diagram arrangements: (a) TE, (b) JT, (c) TEMR, and (d) JTMR.

A brief description of each piece of equipment represented in Figure 1 can be seen in Table 1.

**Table 1:** Description of Equipment in the Process Flow Diagram

nomenclature	description	function
P-01, P-03, P-05, and P-06	plate heat exchangers	heat exchange
P-02 and P-04	cooler heat exchangers	mechanical refrigeration of propane cycle on feed gas stream
P-07 and P-08	chiller heat exchangers	heat exchange between bottom and inlet streams of the separating column (T-02 and T-03)
V-01 and V-02	separator vessel	separation of the gaseous molar fraction (top outlet) from the condensate (bottom outlet)
TE-01	turbo-expander	expands and cools the gas, and generates shaft work that is used in the compressor (C-01) for the TEMR and TE arrangements
JT-01, VLV-01, and JT-02	Joule–Thomson valve	generates fluid expansion for cooling (Joule–Thomson effect)
T-01	demethanizer column	the top gas stream corresponds to sales gas and the bottom liquid stream corresponds to NGL
T-02	de-ethanizer column	the top gas stream corresponds to ethane and the bottom liquid stream corresponds to the stream to feed the T-03 column
T-03	debutanizer column	the top gas stream corresponds to residential LPG and the bottom liquid stream corresponds to C5+
C-01 and C-02	compressor	compression of sales gas volume, C-01 for TEMR and TE arrangements
B-01	pump	NGL pumping
AC-01	air cooler	cooling of the sales gas stream

### 2.2 Basis for Process Simulation

A process simulator was used to model the natural gas production in this study. Peng–Robinson equation of state in the simulator was selected as a fluid package that generates thermodynamic properties for the process. The feed composition is given in Table 2 following the study of Roncada et al.^29^

**Table 2:** Feed Gas Compositions in Molar Percentage

components	mol %
C1	0.7801
C2	0.0966
C3	0.0682
nC3	0.0191
iC4	0.0101
nC5	0.0049
iC5	0.0045
C_6_	0.0033
C_7_^+^	0.0019
N_2_	0.004
CO_2_	0.0073
O_2_	0
richness (C_3_^+^)	11.2%

The operating conditions, design parameters, and efficiency shown in Table 3 were chosen based on Junior et al.,^26^ following the specification limits of the Brazilian National Agency of Petroleum, Natural Gas and Biofuels (ANP) and the Gas Processors Suppliers Association (GPSA).

**Table 3:** Operating Conditions

parameter	value	unit
feed flow rate	3,500,000	m^3^/d
feed and sales gas pressure	7000	kPa g
feed and sales gas temperature	25.00	°C
number of demethanizer column stages	35	
reboiler temperature-demethanizer column	67	°C
number of de-ethanizer column stages	43	
reboiler temperature of de-ethanizer column	90.50	°C
number of stages of debutanizer column	36	
reboiler temperature of debutanizer column	148	°C
efficiency of flow machines (expander, compressor, and pump)	75	%
pressure drops in heat exchangers	20	kPa

The detailed equipment efficiency assumptions made in the simulation are depicted in Table 4.

**Table 4:** Equipment Efficiency and Assumptions

equipment	consideration	reference
compressors	isentropic efficiency: 75%	^30^
pump	isentropic efficiency: 75%	
turbo-expander	isentropic efficiency: 75%	^31^
separating column	adiabatic. theoretical trays efficiency: 100%	^32^
heat exchangers	adiabatic	^18^
coolers	adiabatic	
air cooler	negligible head loss	
separator vessels	adiabatic. No chemical reactions	
valve (Joule–Thomson)	adiabatic	
pipes	adiabatic and without pressure loss	

### 2.3 Exergy Analysis

The exergy analysis conducted in this study considered *T*_0_ = 298.15 K and *P*_0_ = 101.325 kPa as a reference or “dead” state based on the work of Vatani et al.^33^ Table 5 shows the equations representing the exergy analysis used to evaluate the GSP flow diagrams.

**Table 5:** Equations Used to Calculate Exergy for Exergy Balance and Exergy Efficiency^a^

description	equation	
exergy balance	1 $\sum {ṁ}_{in}{ex}_{in}+\sum {\dot{Ex}}_{Q\;in}=\sum {ṁ}_{out}{ex}_{out}+\sum {\dot{Ex}}_{Q\;out}++\sum W+\dot{{Ex}_{D}}$	(1)
exergy equation	2 $\dot{Ex}=ṁ\left[\left(h-h_{0}\right)-T_{0}\left(S-S_{0}\right)\right]$	(2)
exergy efficiency	3 $\varepsilon =\frac{\sum {ṁ}_{out}{ex}_{out}+\sum {\dot{Ex}}_{Q\;out}+\sum W_{out}}{\sum {ṁ}_{in}{ex}_{in}+\sum {\dot{Ex}}_{Q\;in}++\sum W_{in}}=\frac{{\dot{Ex}}_{p}}{{\dot{Ex}}_{F}}=1-\frac{{\dot{Ex}}_{D}}{{\dot{Ex}}_{F}}$	(3)
the total rate of exergy destruction	4 ${\dot{Ex}}_{D}={\dot{Ex}}_{F}-{\dot{Ex}}_{P}+\sum {\dot{Ex}}_{Q\;in}-\sum {\dot{Ex}}_{Q\;out}+\sum W_{in}-\sum W_{out}$	(4)
exergy associated with the product exergy	5 ${\dot{Ex}}_{P}={\dot{Ex}}_{SG}+{\dot{Ex}}_{ethane}+{\dot{Ex}}_{LGP}+{\dot{Ex}}_{NGL}$	(5)

a Where ${\dot{Ex}}_{Q\;in}$ and ${\dot{Ex}}_{Q\;out}$ (kW) correspond to the process heat exergy in and out, respectively; *W* (kW) is work, $\dot{{Ex}_{D}}$ (kW) is the exergy destroyed, ${\dot{Ex}}_{F}$ (kW) is the fuel exergy, ${\dot{Ex}}_{P}$ (kW) is the product exergy, ${\dot{Ex}}_{SG}$ (kW) is the sales gas exergy, ${\dot{Ex}}_{ethane}$ (kW) is the ethane exergy, ${\dot{Ex}}_{LGP}$ (kW) is the LPG exergy, ${\dot{Ex}}_{NGL}$ (kW) is the NGL exergy, ${ṁ}_{in}$ (kg/s) and ${ṁ}_{out}$ (kg/s) are, respectively, the mass flow rates of each equipment, and ex_in_ (kJ/kg) and ex_out_ (kJ/kg) represent the mass exergy of each component. The subscripts in and out represent the input and output streams, respectively, and variables *h* and *S* refer to the enthalpy and entropy, respectively.

The overall exergy efficiency (ε) in eq 3 can be expressed as the difference between the exergy input of the fuel (feed gas), the exergy output of the products (sales gas, ethane, LPG, and NGL), and the exergy outputs and inputs in the form of heat or work.

Based on the work of Gorbani and Amidpour,^34^ the equations related to destroyed exergy and exergy efficiency are specified for each equipment in Table 6.

**Table 6:** Destroyed Exergy and Exergy Efficiency for Unit Operation

equipment	exergy destroyed	eq	exergy efficiency	eq
compressor	6 $\dot{{Ex}_{D}}=\sum {\left(ṁe\right)}_{in}+W-\sum {\left(ṁe\right)}_{out}$	(6)	7 $\varepsilon =\frac{\sum {\left(ṁe\right)}_{in}-\sum {\left(ṁe\right)}_{out}}{W}$	(7)
heat exchanger	$\dot{{Ex}_{D}}=\left[\sum {\left(ṁe\right)}_{1,in,hot}+\sum {\left(ṁe\right)}_{2,in,cold}\right]-\left[\sum {\left(ṁe\right)}_{1,out,cold}+\sum {\left(ṁe\right)}_{2,out,hot}\right]$ 8	(8)	9 $\varepsilon =\frac{\left[\sum {\left(ṁe\right)}_{1,in,hot}-\sum {\left(ṁe\right)}_{2,out,hot}\right]}{\left[\sum {\left(ṁe\right)}_{2,out,cold}-\sum {\left(ṁe\right)}_{2,in,cold}\right]}$	(9)
column	$$\overset{˙}{{Ex}_{D}}=\sum {\left(ṁe\right)}_{in}-\sum {\left(ṁe\right)}_{out}+Q_{r}\left(1-\frac{T_{0}}{T}\right)-***Q_{c}\left(1-\frac{T_{0}}{T}\right)$$ 10	(10)	11 $$\varepsilon =\frac{\sum {\left(ṁe\right)}_{out}+Q_{c}\left(1-\frac{T_{0}}{T}\right)}{\sum {\left(ṁe\right)}_{in}+Q_{r}\left(1-\frac{T_{0}}{T}\right)}$$	(11)
air cooler and heat exchanger cooler	12 $\dot{{Ex}_{D}}=\sum {\left(ṁe\right)}_{in}+Q\left(1-\frac{T_{0}}{T}\right)-\sum {\left(ṁe\right)}_{out}$	(12)	13 $\varepsilon =\frac{Q\left(1-\frac{T_{0}}{T}\right)}{\sum {\left(ṁe\right)}_{in}-\sum {\left(ṁe\right)}_{out}}$	(13)
pump	14 $\dot{{Ex}_{D}}=\sum {\left(ṁe\right)}_{in}+W-\sum {\left(ṁe\right)}_{out}$	(14)	15 $\varepsilon =\frac{\sum {\left(ṁe\right)}_{in}-\sum {\left(ṁe\right)}_{out}}{W}$	(15)
expansion valve	16 $\dot{{Ex}_{D}}=\sum {\left(ṁe\right)}_{in}-\sum {\left(ṁe\right)}_{out}$	(16)	17 $\varepsilon =\frac{e_{out}^{\Delta T}-e_{in}^{\Delta T}}{e_{out}^{\Delta P}-e_{out}^{\Delta P}}e^{\Delta T}=\left[-\int \frac{T-T_{0}}{T}dh\right]e^{\Delta P}=T_{0}\left(s_{o}-S_{in}\right)-\left(h_{o}-h_{in}\right)$	(17)
turbo-expander	18 $\dot{{Ex}_{D}}=\sum {\left(ṁe\right)}_{in}-W-\sum {\left(ṁe\right)}_{out}$	(18)	19 $\varepsilon =\frac{W}{\sum {\left(ṁe\right)}_{in}-\sum {\left(ṁe\right)}_{out}}$	(19)
separator vessel	20 ${\dot{Ex}}_{D}=\sum {\left(ṁe\right)}_{in}-\sum {\left(me\right)}_{out}$	(20)	21 $\varepsilon =\frac{\sum {\left(ṁe\right)}_{out}}{\sum {\left(ṁe\right)}_{in}}$	(21)

Table 7 presents the definitions of fuel and product for each piece of equipment relevant to the exergoeconomic analysis discussed in Section 2.4.

**Table 7:**
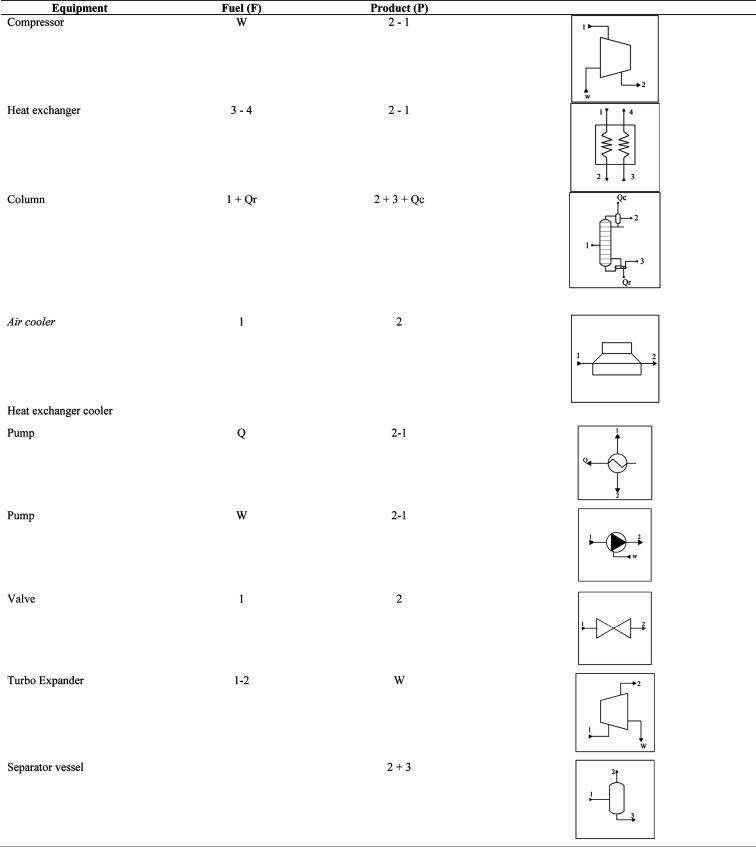
Fuel and Product for Unit Operation^a^

a Where *e* (kJ/kg) is the mass exergy, and $Q\left(1-\frac{T_{0}}{T}\right)$ (kW) is the exergy stream in heat using the Carnot efficiency. The subscripts are *r* for the reboiler exergy and *c* for the condenser exergy.

It is noted that each equipment has a specific equation for the destroyed exergy $\dot{{Ex}_{D}}$ (kW) and ε exergy efficiency (%). The results of each material and energy stream are presented in Tables S1 and S2 (in the Supporting Information).

### 2.4 Exergoeconomic Analysis

The economic model developed by the Electric Power Research Institute was applied.^35^ The Total Revenue Requirement (TRR) was calculated by estimating economic, financial, and operational parameters. The costs related to investment, operation, and maintenance were leveled. The total revenue requirements (total cost of a product) were defined as those generated in a year through product sales to cover all process costs in that specific year and ensure economic operation of the process. These costs were based on two components: carrying charges (CCs) and simple costs (SCs). CC was a general allocation for total investment costs, such as total capital recovery, taxes, and insurance. SCs were used for Fuel Costs (FCs) and Operations and Maintenance Costs (OMCs).

Table 8 presents the economic parameters, operating time, and plant life cycle used to conduct an exergoeconomic analysis. These values are consistent with those used in previous studies on NGL and gas processing technologies, as documented by Mehrpooya and Ansarinasab,^36^ Ghorbani et al.,^37^ and Norouzi et al.,^38^ except for the application of the average annual monetary cost rate (*i*_eff_). In this current research, the Brazilian average annual monetary cost rate (*i*_eff_) was based on the Trading and Economics Web site as of April 30, 2024.^39^

**Table 8:** Economic Parameters, Operating Time, and Plant Life Cycle

parameter	value	reference
Brazilian average annual monetary cost rate (*i*_eff_)	10.8%	^39^
Nominal average escalation rate for operation and maintenance cost (*r*_OMC_)	5%	^36–38^
average nominal escalation rate for fuel (*r*_FC_)	5%	
plant life cycle (BL)	25 years	
total annual operating hours of system at full load	7300 h	

#### 2.4.1 Levelized Costs

The carrying charges (CC_*j*_) and simple costs (OMC_*j*_, FC_*j*_) series for the *j*_th_ year of operation are not a uniform series. Generally, it can be pointed out that fuel costs increase through the years of operation, while investment costs decrease. Using the Capital Recovery Factor (CRF) and devaluation factor, the levelized values for the total annual revenue requirement TRR_L_ were obtained through eq 22

22 $${TRR}_{L}=CRF\cdot \sum_{1}^{BL}\frac{{TRR}_{j}}{{\left(1+i_{eff}\right)}^{j}}$$

where TRR_*j*_ is the total revenue requirement in the *j*_th_ year of operation of the process (US $), *i*_eff_ is the average annual effective depreciation rate, and BL is the economic life cycle of the process (year). The capital recovery factor (CRF) was calculated according to eq 23.

23 $$CRF=\frac{i_{eff}\cdot {\left(1+i_{eff}\right)}^{BL}}{{\left(1+i_{eff}\right)}^{BL}-1}$$

The TRR_*j*_ value was calculated by adding four annual values, which include total capital recovery (TCR), minimum return on investment (ROI), fuel cost (FC) and operation and maintenance cost (OMC) expressed by eq 24.

24 $${TRR}_{j}={TCR}_{j}+{ROI}_{j}+{FC}_{j}+{OMC}_{j}$$

Fuel costs for the process were composed of electricity and heat costs, calculated for the first year of operation by eqs 25 and 27.

25 $${FC}_{0}=\left(C_{w}\cdot Ẇ+C_{q}\cdot \mathrm{Q̇}\right)\cdot \tau$$

26 $$C_{W}=0.18\;US\;\$/kW\;h$$

27 $$C_{q}=0.05\;US\;\$/kW\;h$$

where τ is the annual operating time (h) (Table 7), *Ẇ* is the electrical work (kW), and *Q̇* is the heat (kW) of the process.

Based on the Global Petrol Prices Web site in March 2024, the *C*_W_ and *C*_q_ are used to estimate the costs for electricity and heat, respectively.^40^ The *C*_W_ refers to the average electricity price in the world expressed in US $ per kWh. The *C*_q_ is the average natural gas cost in US $ per kW h. The world average values are used instead of Brazil’s costs to compare GSP fairly with others reported in the literature.

The fuel cost during the *j*_th_ year is calculated with eq 28.

28 $${FC}_{j}={FC}_{0}{\cdot \left(1+r_{FC}\right)}^{j}$$

where *r*_FC_ is the average nominal scale.

Therefore, the levelized fuel cost (FC_L_) (eq 29) was calculated by multiplying the fuel cost at the beginning of the first year with the Constant-Escalation Levelization Factor (CELF).

29 $${FC}_{L}={FC}_{0}\cdot CELF={FC}_{0}\cdot \frac{K_{FC}\cdot \left(1-K_{FC}^{BL}\right)}{\left(1-K_{FC}\right)}\cdot CRF$$

where *K*_FC_ and CELF were calculated by eqs 30 and 31, respectively.

30 $$K_{FC}=\frac{1+r_{FC}}{1+i_{eff}}$$

31 $$CELF=\frac{K_{FC}\left(1-K_{FC}^{BL}\right)}{\left(1-K_{FC}\right)}$$

The terms *r*_FC_ and CRF are the annual escalation rates of fuel cost and capital recovery factor, respectively. Similarly, the levelized cost of operation and maintenance (OMC_L_) was obtained with eq 32.

32 $${OMC}_{L}={OMC}_{0}\cdot CELF={OMC}_{0}\cdot \frac{K_{OMC}\cdot \left(1-K_{OMC}^{BL}\right)}{\left(1-K_{OMC}\right)}\cdot CRF$$

where *K*_OMC_

33 $$K_{OMC}=\frac{1+r_{OMC}}{1+i_{eff}}$$

The term *r*_OMC_ is the annual escalation rate of the operation and maintenance costs. Finally, costs of levelized carrying charges (CC_L_) were calculated by eq 34.

34 $${CC}_{L}={TRR}_{L}-{FC}_{L}-{OMC}_{L}$$

The calculation of the equipment cost rate ( ${Ż}_{K}$ ) (US $/h) was associated with the capital investment ( ${Ż}_{K}^{CI}$ ) and operation and maintenance (*Z*_*K*_OM) cost rates.

35 $${Ż}_{K}={Ż}_{K}^{CI}+{Ż}_{K}^{OM}$$

36 $${Ż}_{K}^{CI}=\frac{{CC}_{L}}{\tau }\frac{{PEC}_{K}}{\sum_{K}{PEC}_{K}}$$

37 $${Ż}_{K}^{OM}=\frac{{OMC}_{L}}{\tau }\frac{{PEC}_{K}}{\sum_{K}{PEC}_{K}}$$

where PEC_*K*_ is the purchased cost of the *K*th equipment (US $) and τ is the operating time (h) for one year.

Relating eqs 36 and 37 obtained the results for capital investments, operation, and maintenance costs for each piece of equipment shown in Table S3 (in the Supporting Information).

38 $${Ż}_{K}={Ż}_{K}^{CI}+{Ż}_{K}^{OM}=\frac{{CC}_{L}+{OMC}_{L}}{\tau }\frac{{PEC}_{K}}{\sum_{K}{PEC}_{K}}$$

As reported by Couper et al.,^41^ the equations for estimating the equipment cost are shown in Table 9. The data of equipment cost of GSP can be found in Table S4 (in the Supporting Information).

**Table 9:** Equations Used for Estimating Equipment Cost

equipment	equipment cost equation	Reference
compressor	$$C=7.90{\left(hp\right)}^{0,62}\;\left(US\;k\$\right)$$	^41^
turbo-expander	$$C=0.38{\left(hp\right)}^{0,81}\;\left(US\;k\$\right)$$	
pump	$$C=F_{M}F_{T}C_{b}\;\left(US\;\$\right)$$	
	$$C_{b}=1.39\exp\left[8.883-0.6019\;\left(\ln Q\sqrt{H}\right)+0.0519{\;\left(\ln Q\sqrt{H}\right)}^{2}\right]$$	
	*Q* in gpm, *H* in ft	
	$F_{T}=\exp\left[b_{1}+b_{2}\;\left(\ln Q\sqrt{H}\right)+b_{3}\;{\left(\ln Q\sqrt{H}\right)}^{2}\right]$ *b*_1_ = 0.0632, *b*_2_ = 0.2744, *b*_3_ = −0.0253, *F*_*M*_ = 1.35 cast steel	
heat exchangers	$C=1.218f_{d}f_{m}f_{p}C_{b}$ (US $)	
	$C_{b}=\exp\left[8.821-030863\;\left(\ln A\right)+0.0681\;{\left(\ln A\right)}^{2}\right]$ *A* in sq ft	
	$f_{d}=\exp\left[-1.1156+0.0906\;\left(\ln A\right)\right]$ $f_{m}=g_{1}+g_{2}\;\left(\ln A\right)$	
	*g*_1_ = 0.8603, *g*_2_ = 0.23296	
	$$f_{p}=1.1400+0.12088\;\left(\ln A\right)$$	
separator vessel	$$C=F_{M}C_{b}+C_{a}\;\left(US\;\$\right)$$	
	$$C_{b}=1.218\exp\left[9.100-0.2889\;\left(\ln W\right)+0.04333{\;\left(\ln W\right)}^{2}\right]$$	
	*W* in lbf.ft	
	$$C_{a}=300D^{0.7396}L^{0.7066}$$	
	*D* and *L* in ft	
column	$$C=1.218\left[f_{1}C_{b}+Nf_{2}f_{3}f_{4}C_{t}+C_{pt}\right]\;\left(US\;\$\right)$$	
	$$C_{b}=1.218\exp\left[7.123+01478\;\left(\ln W\right)+0.02488{\;\left(\ln W\right)}^{2}\ln\left(\frac{T_{b}}{T_{c}}\right)\right]$$	
	$C_{t}=457.7\exp\left(0.1739D\right)$ *N* = number of stages	
	*C*_p1_ = 249.6*D* ^0.6332^ *L* ^0.8016^ *f*_1_ and *f*_2_ material factor	
	*f*_3_ stage type factor	
	*f*_2_ stage quantity factor	

Levelized fuel cost rate related to the costs provided for the whole system can be calculated as follows

39 $$\dot{C_{F}}=\frac{{FC}_{L}}{\tau }$$

#### 2.4.2 Cost Balance Equations

The equipment exergy cost rate balance was defined as the product exergy cost rate equal to the sum of the fuel exergy cost rate and equipment cost rate (eq 40).

40 $${Ċ}_{p}={Ċ}_{F}+{Ż}^{tot}$$

where ${Ż}^{tot}$ (US $/h) is the equipment cost rate.

The rate of the *i*_th_ material stream, ${Ċ}_{i}$ , was expressed as the product of the exergy stream ${Ė}_{i}$ and average cost per exergy unit *c*_*i*_, according to eqs 41, 42, and 43.

41 $${Ċ}_{i}=c_{i}\cdot {Ė}_{i}$$

42 $${Ċ}_{W}=c_{W}\cdot Ẇ$$

43 $${Ċ}_{q}=c_{q}\cdot \mathrm{Q̇}$$

where ${Ċ}_{i}$ (US $/h) is the exergy cost rate, ${Ė}_{i}$ (kW) is the exergy, *c*_*i*_ ($/GJ) is the average unit exergy cost of the *i*_th_ stream, *c*_w_ (US $/kW h) and *c*_q_ (US $/kW h) is the average unit exergy cost for electrical power and heat. The exergy and unit cost of material streams for each arrangement can be found in Table S5 (in the Supporting Information).

The cost balance for each piece of process equipment was calculated according to eqs 44 and 45.

44 $$\sum_{in}{Ċ}_{i\;in,K}+{Ċ}_{q,K}+\dot{Z_{K}=\sum_{out}{Ċ}_{i\;out,K}+{Ċ}_{w,K}}$$

45 $$\sum_{in}{\left(c_{i}{Ė}_{i}\right)}_{in\;K}+{Ż}_{K}^{CI}+{Ż}_{K}^{OM}=\sum_{out}{\left(c_{i}{Ė}_{i}\right)}_{out\;K}$$

where ${Ċ}_{K}$ ($/h) represents the exergy cost for the exergy flows associated with the *K* ^th^ equipment $\left({{Ċ}_{i\;in,K},Ċ}_{i\;out,K},{Ċ}_{w,K}eC_{q,K}\right)$ .

The cost rate of exergy for the *K* ^th^ equipment resulted in a set of linear eq (eq 46).

46 $$\left[{Ė}_{K}\right]\times \left[c_{K}\right]=\left[{Ż}_{K}\right]$$

where $\left[{Ė}_{K}\right]$ , [*c*_*K*_], and $\left[{Ż}_{K}\right]$ are the exergy intensity matrix, unknown exergy cost vector, and coefficient vector, respectively.

The costs and auxiliary equations for each process diagram arrangement are shown in Tables 10–13.

**Table 10:** Cost Equations for the TE Process Diagram Arrangement

equipment	equation	auxiliary equation
P-01	${Ċ}_{gas}+{Ċ}_{15}+{Ż}_{P‐01}={Ċ}_{1}+{Ċ}_{16}$	$\frac{{Ċ}_{1}-{Ċ}_{gas}}{{Ė}_{1}-{Ė}_{gas}}=\frac{{Ċ}_{16}-{Ċ}_{15}}{{Ė}_{16}-{Ė}_{15}}$
P-03	${Ċ}_{1}+{Ċ}_{14}+{Ż}_{P‐03}={Ċ}_{2}+{Ċ}_{15}$	$\frac{{Ċ}_{2}-{Ċ}_{1}}{{Ė}_{2}-{Ė}_{1}}=\frac{{Ċ}_{15}-{Ċ}_{14}}{{Ė}_{15}-{Ė}_{14}}$
P-05	${Ċ}_{2}+{Ċ}_{13}+{Ż}_{P‐05}={Ċ}_{3}+{Ċ}_{14}$	$\frac{{Ċ}_{3}-{Ċ}_{2}}{{Ė}_{3}-{Ė}_{2}}=\frac{{Ċ}_{14}-{Ċ}_{13}}{{Ė}_{14}-{Ė}_{13}}$
P-06	${Ċ}_{7}+{Ċ}_{12}+{Ż}_{P‐06}={Ċ}_{10}+{Ċ}_{13}$	$\frac{{Ċ}_{10}-{Ċ}_{7}}{{Ė}_{10}-{Ė}_{7}}=\frac{{Ċ}_{13}-{Ċ}_{12}}{{Ė}_{13}-{Ė}_{12}}$
V-01	${Ċ}_{3}+{Ż}_{V‐01}={Ċ}_{4}+{Ċ}_{5}$	$\frac{{Ċ}_{4}}{{Ė}_{5}}=\frac{{Ċ}_{5}}{{Ė}_{5}}$
V-02	${Ċ}_{15}+{Ż}_{V‐02}={Ċ}_{17}$	
JT-02	${Ċ}_{10}={Ċ}_{11}$	
VLV-01	${Ċ}_{4}={Ċ}_{6}$	
TE-01	${Ċ}_{8}+{Ż}_{TE‐01}={Ċ}_{9}+{Ċ}_{W\;TE‐01}$	
T-01	${Ċ}_{6}+{Ċ}_{9}+{Ċ}_{11}+{Ċ}_{q\;ET‐01}+{Ż}_{T‐01}={Ċ}_{NGL}+{Ċ}_{12}$	$\frac{{Ċ}_{12}}{{Ė}_{12}}=\frac{{Ċ}_{LGN}}{{Ė}_{LGN}}$
T-02	${Ċ}_{22}+{Ċ}_{q\;ET‐02}+{Ċ}_{qQ\;T‐02}+{Ż}_{T‐02}={Ċ}_{C2}+{Ċ}_{23}$	$\frac{{Ċ}_{C_{2}}}{{Ė}_{C_{2}}}=\frac{{Ċ}_{23}}{{Ė}_{23}}$
T-03	${Ċ}_{25}+{Ċ}_{q\;ET‐03}+{Ċ}_{qQ\;T‐03}+{Ż}_{T‐03}={Ċ}_{LGP}+{Ċ}_{26}$	$\frac{{Ċ}_{GLP}}{{Ė}_{GLP}}=\frac{{Ċ}_{26}}{{Ė}_{26}}$
C-01	${Ċ}_{17}+{Ċ}_{W\;C‐01}+{Ż}_{C‐01}={Ċ}_{19}$	
C-02	${Ċ}_{19}+{Ċ}_{W\;C‐02}+{Ż}_{C‐02}={Ċ}_{20}$	
AC-01	${Ċ}_{20}+{Ż}_{AC‐01}={Ċ}_{SG}$	
B-01	${Ċ}_{NGL}+{Ċ}_{W\;B‐01}+{Ż}_{B‐01}={Ċ}_{21}$	
P-07	${Ċ}_{21}+{Ċ}_{23}+{Ż}_{P‐07}={Ċ}_{22}+{Ċ}_{24}$	$\frac{{Ċ}_{22}-{Ċ}_{21}}{{Ė}_{22}-{Ė}_{21}}=\frac{{Ċ}_{24}-{Ċ}_{23}}{{Ė}_{24}-{Ė}_{23}}$
P-08	${Ċ}_{24}+{Ċ}_{26}+{Ż}_{P‐08}={Ċ}_{25}+{Ċ}_{C_{5}^{+}}$	$\frac{{Ċ}_{25}-{Ċ}_{24}}{{Ė}_{25}-{Ė}_{24}}=\frac{{Ċ}_{C_{5}^{+}}-{Ċ}_{26}}{{Ċ}_{C_{5}^{+}}-{Ė}_{26}}$
TEE-01	${Ċ}_{5}={Ċ}_{7}+{Ċ}_{8}$	$\frac{{Ċ}_{7}}{{Ė}_{7}}=\frac{{Ċ}_{8}}{{Ė}_{8}}$

**11:** Cost Equations for the JT Process Diagram Arrangement

Equipment	Equation	Auxiliary Equation
P-01	${Ċ}_{gas}+{Ċ}_{15}+{Ż}_{P‐01}={Ċ}_{1}+{Ċ}_{16}$	$\frac{{Ċ}_{1}-{Ċ}_{gas}}{{Ė}_{1}-{Ė}_{gas}}=\frac{{Ċ}_{16}-{Ċ}_{15}}{{Ė}_{16}-{Ė}_{15}}$
P-03	${Ċ}_{1}+{Ċ}_{14}+{Ż}_{P‐03}={Ċ}_{2}+{Ċ}_{15}$	$\frac{{Ċ}_{2}-{Ċ}_{1}}{{Ė}_{2}-{Ė}_{1}}=\frac{{Ċ}_{15}-{Ċ}_{14}}{{Ė}_{15}-{Ė}_{14}}$
P-05	${Ċ}_{2}+{Ċ}_{13}+{Ż}_{P‐05}={Ċ}_{3}+{Ċ}_{14}$	$\frac{{Ċ}_{3}-{Ċ}_{2}}{{Ė}_{3}-{Ė}_{2}}=\frac{{Ċ}_{14}-{Ċ}_{13}}{{Ė}_{14}-{Ė}_{13}}$
P-06	${Ċ}_{7}+{Ċ}_{12}+{Ż}_{P‐06}={Ċ}_{10}+{Ċ}_{13}$	$\frac{{Ċ}_{10}-{Ċ}_{7}}{{Ė}_{10}-{Ė}_{7}}=\frac{{Ċ}_{13}-{Ċ}_{12}}{{Ė}_{13}-{Ė}_{12}}$
V-01	${Ċ}_{3}+{Ż}_{V‐01}={Ċ}_{5}+{Ċ}_{4}$	$\frac{{Ċ}_{5}}{{Ė}_{5}}=\frac{{Ċ}_{4}}{{Ė}_{4}}$
V-02	${Ċ}_{16}+{Ż}_{V‐02}={Ċ}_{17}$	
JT - 02	${Ċ}_{10}={Ċ}_{11}$	
VLV-01	${Ċ}_{4}={Ċ}_{6}$	
JT-01	${Ċ}_{8}+{Ż}_{JT‐01}={Ċ}_{9}$	
T-01	${Ċ}_{6}+{Ċ}_{9}+{Ċ}_{11}+{Ċ}_{q\;ET‐01}+{Ż}_{T‐01}={Ċ}_{NGL}+{Ċ}_{12}$	$\frac{{Ċ}_{12}}{{Ė}_{12}}=\frac{{Ċ}_{LGN}}{{Ė}_{LGN}}$
T-02	${Ċ}_{21}+{Ċ}_{q\;ET‐02}+{Ċ}_{q\;QT‐02}+{Ż}_{T‐02}={Ċ}_{C_{2}}+{Ċ}_{22}$	$\frac{{Ċ}_{C_{2}}}{{Ė}_{C_{2}}}=\frac{{Ċ}_{22}}{{Ė}_{22}}$
T-03	${Ċ}_{24}+{Ċ}_{q\;ET‐03}+{Ċ}_{qQ\;T‐03}+{Ż}_{T‐03}={Ċ}_{LPG}+{Ċ}_{25}$	$\frac{{Ċ}_{GLP}}{{Ė}_{GLP}}=\frac{{Ċ}_{25}}{{Ė}_{25}}$
C-02	${Ċ}_{17}+{Ċ}_{W\;C‐02}+{Ż}_{C‐02}={Ċ}_{19}$	
AC-01	${Ċ}_{19}+{Ż}_{AC‐01}={Ċ}_{SG}$	
B-01	${Ċ}_{NGL}+{Ċ}_{W\;B‐01}+{Ż}_{B‐01}={Ċ}_{20}$	
P-07	${Ċ}_{20}+{Ċ}_{22}+{Ż}_{P‐07}={Ċ}_{21}+{Ċ}_{23}$	$\frac{{Ċ}_{21}-{Ċ}_{20}}{{Ė}_{21}-{Ė}_{20}}=\frac{{Ċ}_{23}-{Ċ}_{22}}{{Ė}_{23}-{Ė}_{22}}$
P-08	${Ċ}_{23}+{Ċ}_{25}+{Ż}_{P‐08}={Ċ}_{24}+{Ċ}_{C_{5}^{+}}$	$\frac{{Ċ}_{24}-{Ċ}_{23}}{{Ė}_{24}-{Ė}_{23}}=\frac{{Ċ}_{C_{5}^{+}}-{Ċ}_{25}}{{Ċ}_{C_{5}^{+}}-{Ė}_{25}}$
TEE-01	${Ċ}_{5}={Ċ}_{8}+{Ċ}_{7}$	$\frac{{Ċ}_{8}}{{Ė}_{8}}=\frac{{Ċ}_{7}}{{Ė}_{7}}$

**12:** Cost Equations for the TEMR Process Diagram Arrangement

equipment	equation	auxiliary equation
P-01	${Ċ}_{gas}+{Ċ}_{17}+{Ż}_{P‐01}={Ċ}_{1}+{Ċ}_{18}$	$\frac{{Ċ}_{1}-{Ċ}_{gas}}{{Ė}_{1}-{Ė}_{gas}}=\frac{{Ċ}_{18}-{Ċ}_{17}}{{Ė}_{18}-{Ė}_{17}}$
P-02	${Ċ}_{1}+{Ż}_{P‐02}={Ċ}_{2}+{Ċ}_{Q\;P‐02}$	
P-03	${Ċ}_{2}+{Ċ}_{16}+{Ż}_{P‐03}={Ċ}_{3}+{Ċ}_{17}$	$\frac{{Ċ}_{3}-{Ċ}_{2}}{{Ė}_{3}-{Ė}_{2}}=\frac{{Ċ}_{17}-{Ċ}_{16}}{{Ė}_{17}-{Ė}_{16}}$
P-04	${Ċ}_{3}+{Ż}_{P‐04}={Ċ}_{4}+{Ċ}_{QP‐04}$	
P-05	${Ċ}_{4}+{Ċ}_{15}+{Ż}_{P‐05}={Ċ}_{5}+{Ċ}_{16}$	$\frac{{Ċ}_{5}-{Ċ}_{4}}{{Ė}_{5}-{Ė}_{4}}=\frac{{Ċ}_{16}-{Ċ}_{15}}{{Ė}_{16}-{Ė}_{15}}$
P-06	${Ċ}_{9}+{Ċ}_{14}+{Ż}_{P‐06}={Ċ}_{10}+{Ċ}_{15}$	$\frac{{Ċ}_{10}-{Ċ}_{9}}{{Ė}_{10}-{Ė}_{9}}=\frac{{Ċ}_{15}-{Ċ}_{14}}{{Ė}_{15}-{Ė}_{14}}$
V-01	${Ċ}_{5}+{Ż}_{V‐01}={Ċ}_{8}+{Ċ}_{6}$	$\frac{{Ċ}_{6}}{{Ė}_{6}}=\frac{{Ċ}_{8}}{{Ė}_{8}}$
V-02	${Ċ}_{18}+{Ż}_{V‐02}={Ċ}_{19}$	
JT-02	${Ċ}_{10}={Ċ}_{11}$	
VLV-01	${Ċ}_{6}={Ċ}_{7}$	
TE-01	${Ċ}_{12}+{Ż}_{TE‐01}={Ċ}_{13}+{Ċ}_{W\;TE‐01}$	
T-01	${Ċ}_{7}+{Ċ}_{11}+{Ċ}_{13}+{Ċ}_{qE\;T‐01}+{Ż}_{T‐01}={Ċ}_{NGL}+{Ċ}_{14}$	$\frac{{Ċ}_{14}}{{Ė}_{14}}=\frac{{Ċ}_{LGN}}{{Ė}_{LGN}}$
T-02	${Ċ}_{24}+{Ċ}_{qE\;T‐02}+{Ċ}_{qQ\;T‐02}+{Ż}_{T‐02}={Ċ}_{C_{2}}+{Ċ}_{25}$	$\frac{{Ċ}_{C_{2}}}{{Ė}_{C_{2}}}=\frac{{Ċ}_{25}}{{Ė}_{25}}$
T-03	${Ċ}_{27}+{Ċ}_{qE\;T‐03}+{Ċ}_{qQ\;T‐03}+{Ż}_{T‐03}={Ċ}_{LPG}+{Ċ}_{28}$	$\frac{{Ċ}_{GLP}}{{Ė}_{GLP}}=\frac{{Ċ}_{28}}{{Ė}_{28}}$
C-01	${Ċ}_{19}+{Ċ}_{W\;C‐01}+{Ż}_{C‐01}={Ċ}_{21}$	
C-02	${Ċ}_{21}+{Ċ}_{WC‐02}+{Ż}_{C‐02}={Ċ}_{22}$	
AC-01	${Ċ}_{22}+{Ż}_{AC‐01}={Ċ}_{SG}$	
B-01	${Ċ}_{NGL}+{Ċ}_{WB‐01}+{Ż}_{B‐01}={Ċ}_{23}$	
P-07	${Ċ}_{23}+{Ċ}_{25}+{Ż}_{P‐07}={Ċ}_{24}+{Ċ}_{26}$	$\frac{{Ċ}_{24}-{Ċ}_{23}}{{Ė}_{24}-{Ė}_{23}}=\frac{{Ċ}_{26}-{Ċ}_{25}}{{Ė}_{26}-{Ė}_{25}}$
P-08	${Ċ}_{26}+{Ċ}_{28}+{Ż}_{P‐08}={Ċ}_{27}+{Ċ}_{C_{5}^{+}}$	$\frac{{Ċ}_{27}-{Ċ}_{26}}{{Ė}_{27}-{Ė}_{26}}=\frac{{Ċ}_{C_{5}^{+}}-{Ċ}_{28}}{{Ċ}_{C_{5}^{+}}-{Ė}_{28}}$
TEE-01	${Ċ}_{8}={Ċ}_{9}+{Ċ}_{12}$	$\frac{{Ċ}_{9}}{{Ė}_{9}}=\frac{{Ċ}_{12}}{{Ė}_{12}}$

**Table 13:** Cost Equations for the JTMR Process Diagram Arrangement

equipment	equation	auxiliary equation
P-01	${Ċ}_{gas}+{Ċ}_{17}+{Ż}_{P‐01}={Ċ}_{1}+{Ċ}_{18}$	$\frac{{Ċ}_{1}-{Ċ}_{gas}}{{Ė}_{1}-{Ė}_{gas}}=\frac{{Ċ}_{18}-{Ċ}_{17}}{{Ė}_{18}-{Ė}_{17}}$
P-02	${Ċ}_{1}+{Ż}_{P‐02}={Ċ}_{2}+{Ċ}_{Q\;P‐02}$	
P-03	${Ċ}_{2}+{Ċ}_{16}+{Ż}_{s‐03}={Ċ}_{3}+{Ċ}_{17}$	$\frac{{Ċ}_{3}-{Ċ}_{2}}{{Ė}_{3}-{Ė}_{2}}=\frac{{Ċ}_{17}-{Ċ}_{18}}{{Ė}_{17}-{Ė}_{18}}$
P-04	${Ċ}_{3}+{Ż}_{P‐04}={Ċ}_{4}+{Ċ}_{Q\;P‐04}$	
P-05	${Ċ}_{4}+{Ċ}_{15}+{Ż}_{P‐05}={Ċ}_{5}+{Ċ}_{16}$	$\frac{{Ċ}_{5}-{Ċ}_{4}}{{Ė}_{5}-{Ė}_{4}}=\frac{{Ċ}_{16}-{Ċ}_{15}}{{Ė}_{16}-{Ė}_{15}}$
P-06	${Ċ}_{9}+{Ċ}_{14}+{Ż}_{P‐06}={Ċ}_{11}+{Ċ}_{15}$	$\frac{{Ċ}_{11}-{Ċ}_{9}}{{Ė}_{11}-{Ė}_{9}}=\frac{{Ċ}_{15}-{Ċ}_{14}}{{Ė}_{15}-{Ė}_{14}}$
V-01	${Ċ}_{5}+{Ż}_{V‐01}={Ċ}_{7}+{Ċ}_{6}$	$\frac{{Ċ}_{7}}{{Ė}_{7}}=\frac{{Ċ}_{6}}{{Ė}_{6}}$
V-02	${Ċ}_{18}+{Ż}_{V‐02}={Ċ}_{19}$	
JT-02	${Ċ}_{11}={Ċ}_{13}$	
VLV-01	${Ċ}_{6}={Ċ}_{8}$	
JT-01	${Ċ}_{10}+{Ż}_{JT‐01}={Ċ}_{12}$	
T-01	${Ċ}_{8}+{Ċ}_{12}+{Ċ}_{13}+{Ċ}_{qE\;T‐01}+{Ż}_{T‐01}={Ċ}_{LGN}+{Ċ}_{14}$	$\frac{{Ċ}_{14}}{{Ė}_{14}}=\frac{{Ċ}_{LGN}}{{Ė}_{LGN}}$
T-02	${Ċ}_{23}+{Ċ}_{qE\;T‐02}+{Ċ}_{qQ\;T‐02}+{Ż}_{T‐02}={Ċ}_{C_{2}}+{Ċ}_{24}$	$\frac{{Ċ}_{C_{2}}}{{Ė}_{C_{2}}}=\frac{{Ċ}_{24}}{{Ė}_{24}}$
T-03	${Ċ}_{26}+{Ċ}_{qE\;T‐03}+{Ċ}_{qQ\;T‐03}+{Ż}_{T‐03}={Ċ}_{LPG}+{Ċ}_{27}$	$\frac{{Ċ}_{GLP}}{{Ė}_{GLP}}=\frac{{Ċ}_{27}}{{Ė}_{27}}$
C-02	${Ċ}_{19}+{Ċ}_{W\;C‐02}+{Ż}_{C‐02}={Ċ}_{21}$	
AC-01	${Ċ}_{21}+{Ż}_{AC‐01}={Ċ}_{SG}$	
B-01	${Ċ}_{NGL}+{Ċ}_{WB‐01}+{Ż}_{B‐01}={Ċ}_{22}$	
P-07	${Ċ}_{22}+{Ċ}_{24}+{Ż}_{P‐07}={Ċ}_{23}+{Ċ}_{25}$	$\frac{{Ċ}_{23}-{Ċ}_{22}}{{Ė}_{23}-{Ė}_{22}}=\frac{{Ċ}_{25}-{Ċ}_{24}}{{Ė}_{25}-{Ė}_{24}}$
P-08	${Ċ}_{25}+{Ċ}_{27}+{Ż}_{P‐08}={Ċ}_{26}+{Ċ}_{C_{5}^{+}}$	$\frac{{Ċ}_{26}-{Ċ}_{25}}{{Ė}_{26}-{Ė}_{25}}=\frac{{Ċ}_{C_{5}^{+}}-{Ċ}_{27}}{{Ċ}_{C_{5}^{+}}-{Ė}_{27}}$
TEE-01	${Ċ}_{7}={Ċ}_{10}+{Ċ}_{9}$	$\frac{{Ċ}_{10}}{{Ė}_{10}}=\frac{{Ċ}_{9}}{{Ė}_{9}}$

#### 2.4.3 Exergoeconomic Variables

Based on the definition of fuel and product of equipment as shown in Table 6, the fuel cost rate ( ${Ċ}_{F}$ ) and product cost rate ( ${Ċ}_{P}$ ) can be calculated. The average fuel cost ratio for the *K*_th_ equipment in the process ${Ċ}_{F,K}$ shows the average unit fuel cost per exergy as follows in eq 47.

47 $$c_{F,K}=\frac{{Ċ}_{F,K}}{{Ė}_{F,K}}$$

Similarly, the exergy product cost $\left({Ċ}_{P,k}\right)$ for the *K*_th_ equipment is defined according to the equation

48 $$c_{P,K}=\frac{{Ċ}_{P,K}}{{Ė}_{P,K}}$$

where ${Ċ}_{P}$ is the product cost rate (US $/h) and ${Ė}_{P,K}$ is the product exergy rate (kW) for the *K*_th_ equipment.

The exergy destruction cost rate ( ${Ċ}_{D,K}$ ) is related to the exergy destruction, ${Ė}_{D,K}$ , and for the *K*_th_ equipment is considered a hidden cost that is revealed in the exergoeconomic analysis. This cost can be calculated by the extra fuel cost required to compensate for the exergy destruction, at which the average cost of generating the product remains practically constant

49 $${Ċ}_{D,K}=c_{F,K}{Ė}_{D,K}$$

In addition, an overall energy destruction cost to the process arrangements can be expressed in terms of Fuel ( ${Ċ}_{F,process}$ ) *e* total exergy destruction ( ${Ė}_{D,process}$ ) as the following equation

50 $${Ċ}_{D,process}={Ċ}_{F,process}\cdot {Ė}_{D,process}$$

New parameters (*r*_*K*_, *f*_*K*_) were defined based on the results provided by the exergoeconomic analysis. The *r*_*K*_ shows the relative increase in the average cost per exergy unit between the fuel and the component product (eq 50).

51 $$r_{K}=\frac{c_{P,K}-c_{F,K}}{C_{F,K}}$$

The exergoeconomic factor, *f*_*K*_ (%), was defined as the ratio of the investment cost of the K_th_ equipment to the total cost ( ${Ż}_{K}+{Ċ}_{D,k}$ ). This parameter shows the relative importance between nonexergy-related costs and exergy-related costs.

52 $$f_{K}=\frac{{ż}_{K}}{{Ż}_{K}+{Ċ}_{D,K}}$$

## 3 Results and Discussion

### 3.1 Exergy Analysis

Figure 2 illustrates the *P*–*e* diagram. State E1 represents the equilibrium state of the gas at the process inlet (7000 kPa g and 25 °C) for each process arrangement. State E2 refers to the state of the feed stream after it has passed through the heat exchangers and before it undergoes expansion in the Turbo-Expander (TE and TEMR) or the Joule–Thompson valve (JT and JTMR). E3 indicates the stream after expansion whether in the Turbo-Expander (TE and TEMR) or the Joule–Thompson valve (JT and JTMR). The displacement of points E1 and E2 is a result of thermal integration of the plate heat exchangers. For TE and JT arrangements, the feed stream is cooled by integrating plate heat exchangers with the sales gas (SG) stream, leaving the top of the demethanizer column. In contrast to the TE and JT, TEMR and JTMR arrangements also incorporate mechanical refrigeration cycles alongside thermal integration. When comparing these process arrangements, it is observed that JT and TE present a small variation in mass exergy since they involve only the feed stream cooling through heat exchangers. Although TE and JT show small changes in mass exergy, TEMR achieves a great variation in mass exergy (kW/kg) between states E2 (before expansion) and E3 (after expansion) (Figure 2).

**Figure 2:**
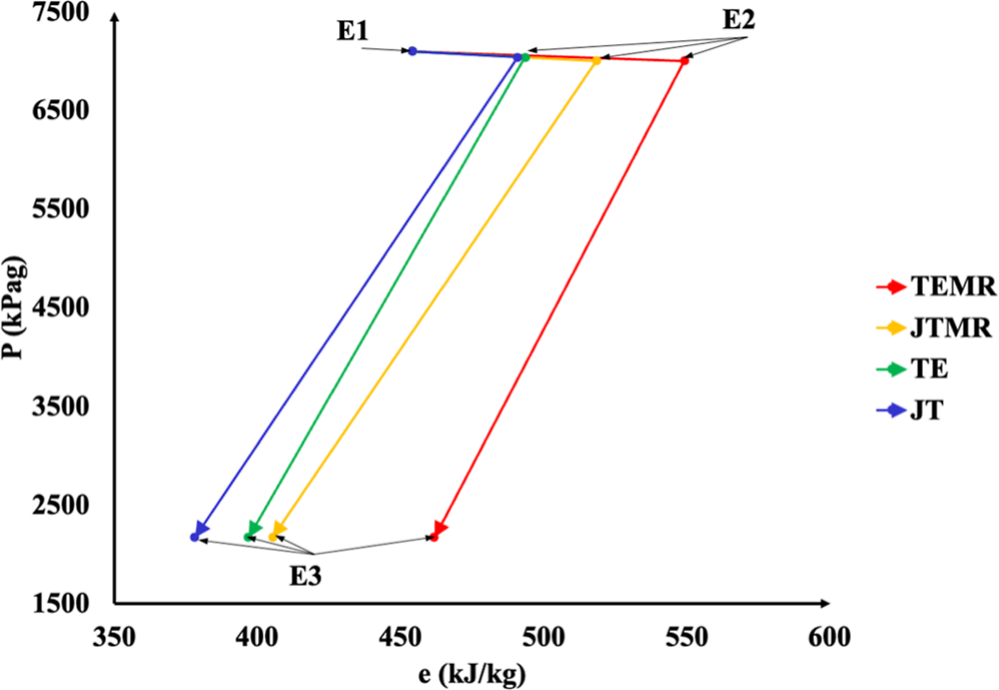
Pressure vs mass exergy diagram.

Figure 3 illustrates the inclination of the line in the P-Exergy diagram. In Figure 3, it can be observed that for the same state, E2, TE, and JT arrangements contain larger amounts of exergy associated with the gas flow from the top of the separator vessel V-01, which is greater than that of the TEMR and JTMR. This results from the cooling of the feed gas. The lower the stream cooling is, the higher the gas temperature at the inlet of the separator vessel, thus producing a larger gas rate at the outlet of the top of V-01, as observed in TE and JT. The changes in exergy between states E2 and E3 are attributed to the process irreversibility associated with the expansion equipment used in the process, specifically the JT-01 valve and the TE-01 Turbo-Expander. In JT and JTMR, a parcel of exergy is destroyed due to changes in entropy, whereas in TE and TEMR, exergy is not only destroyed but partially transformed into shaft work. This demonstrates the TE-enhanced expansion efficiency as it maintains consistent pressure changes between states E2 and E3.

**Figure 3:**
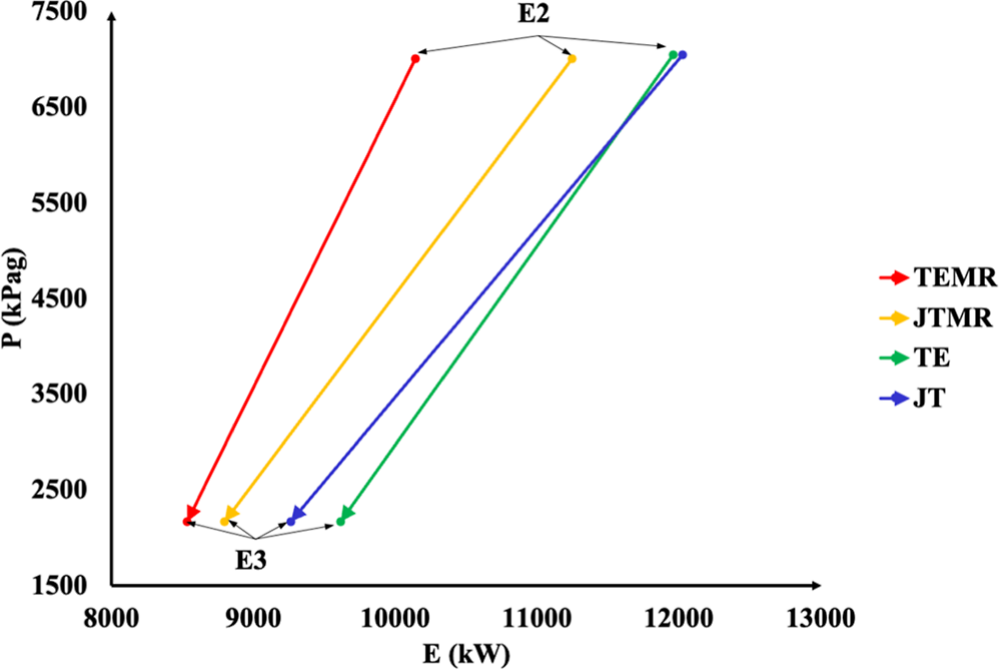
Pressure vs exergy diagram.

Based on the process stages depicted in Figures 2 and 3, pressure variations play a critical role in the thermodynamic performance of plate heat exchangers, expansion devices, and separation units. Plate heat exchangers, which are widely employed for gas cooling and energy recovery, are particularly sensitive to pressure differentials. Excessive pressure drops increase compression energy requirements and lead to exergy destruction, while insufficient pressure differences diminish the heat transfer effectiveness. Therefore, maintaining optimal pressure gradients is essential for maximizing thermal exchange and minimizing irreversibilities.

Expansion devices, such as Turbo-Expanders and Joule–Thomson valves, utilize pressure reductions to induce gas cooling and facilitate NGL condensation. Turbo-Expanders offer higher exergy efficiency by recovering mechanical work, whereas JT valves result in greater exergy losses due to isenthalpic expansion. In separation units, such as demethanizers, pressure reduction enhances hydrocarbon condensation; however, it simultaneously increases the reboiler duty, leading to substantial exergy destruction. Therefore, precise pressure control across all stages is fundamental to improving energy efficiency, minimizing exergy losses, and enabling economically and environmentally sustainable plant operations.

After expansion, the gas stream is separated by distillation into three stages: demethanizer, de-ethanizer, and debutanizer columns. For each arrangement of the process diagram, Figure 4 illustrates the exergy destruction (*D*), the reboiler heat (RQ), and the temperature of the inlet stream from the expansion (*T*_E,in_) of the demethanizer column (T-01). The TEMR achieves lower temperatures Among the arrangements, TEMR had the highest exergy destruction at approximately 2684.52 kW, about 2.6 times greater than that of the JT, which had the lowest destruction at 1019.11 kW at the column inlet, mainly due to the additional mechanical refrigeration, thus requiring the reboiler to produce more heat (QR) to reach the bottom product specification temperature (NGL) of 67 °C.

**Figure 4:**
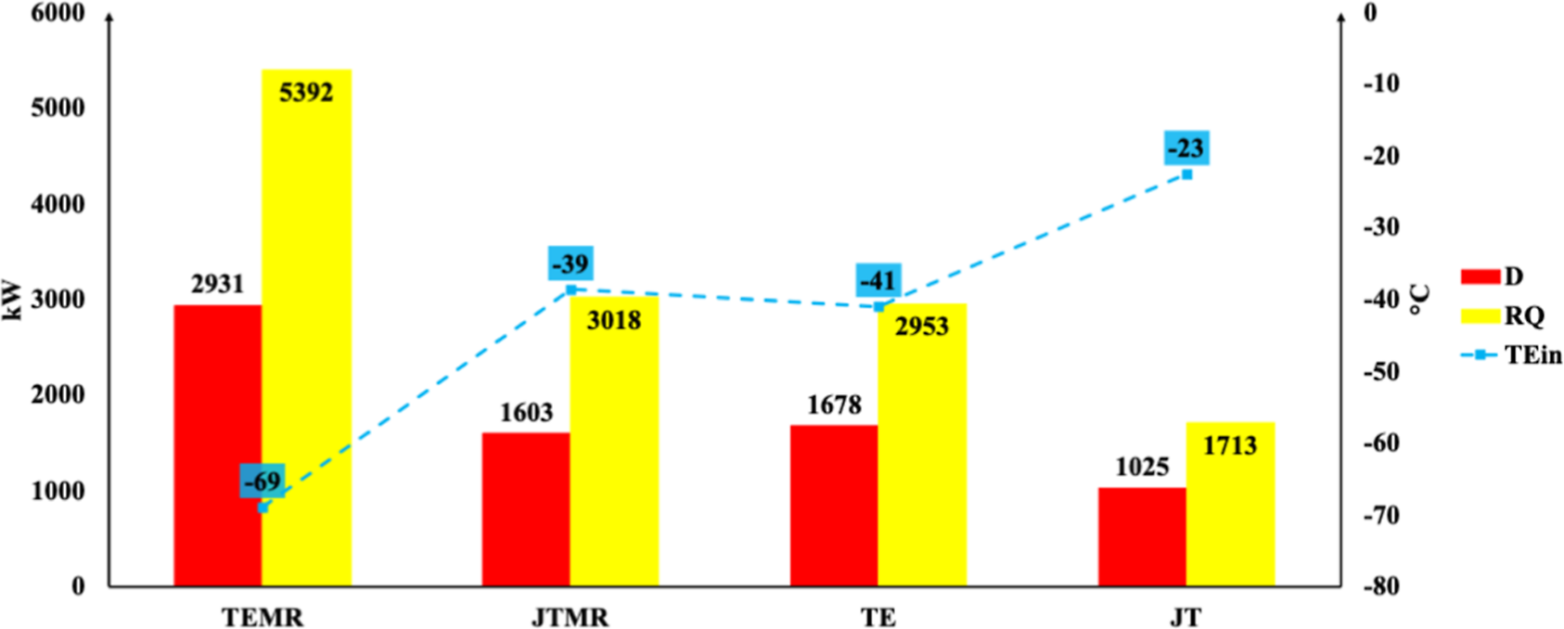
Exergy analysis in the T-01 demethanizer column. Destruction of exergy (*D*), reboiler heat (QR), and expansion stream inlet temperature (*T*_E,in_).

Analyzing the results in Figure 4, it can be stated that exergy destruction is associated with the temperature differences between the inlet and outlet bottom product streams. The larger the temperature difference between the column inlet and the bottom (NGL) temperature, the greater the destruction. Consequently, higher NGL recovery efficiencies lead to increased exergy destruction. Therefore, the TEMR has the highest recovery efficiency (96%), while the JT has the lowest (62%). These findings align with the recovery efficiency results reported by Amaral Junior et al.^26^ The authors observed that the TEMR process achieves the highest recovery rate (96%) compared to that of the JT process (62%). In terms of composition, Shin et al.^42^ reported that streams rich in C_3_^+^ components exhibit greater exergy destruction, which is challenging to reduce due to the high energy demand associated with column operations.

For the demethanizer (T-01) column, Figures 5 and 6 show a regression analysis conducted to clarify the relationship between exergy destruction (*D*) and inlet T-01 temperature (*T*_ein_) and that between D and reboiler heat (QR). The results indicate a negative correlation between the stream inlet temperature (*T*_E,in_) and exergy destruction (*D*) (*R* ^2^ = 0.93), suggesting that lower inlet temperatures significantly increase the irreversibility due to heightened reboiler heat. Therefore, the introduction of mechanical refrigeration (TEMR and JTMR configurations) substantially reduces *T*_E,in_, increases QR demands, and thereby drives exergy destruction upward.

**Figure 5:**
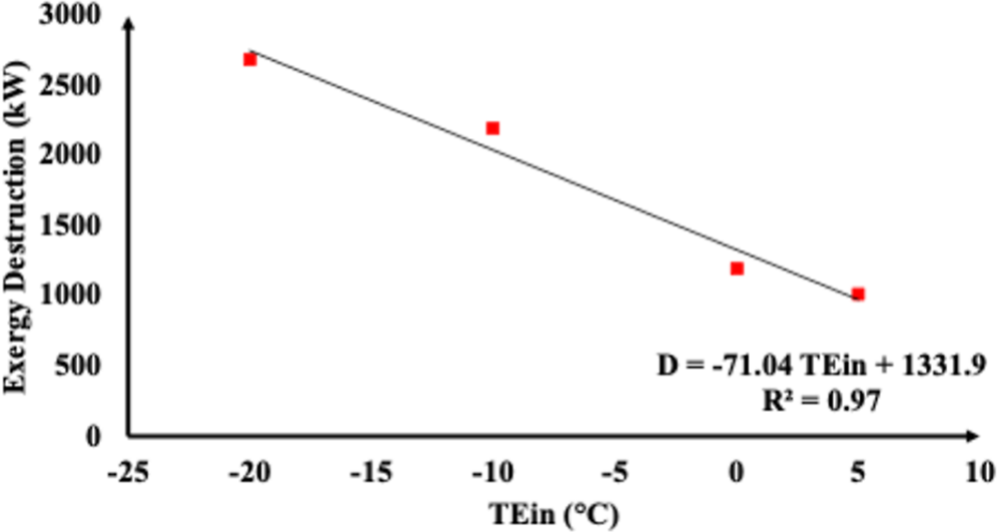
Exergy destruction as a function of inlet T-01 temperature.

**Figure 6:**
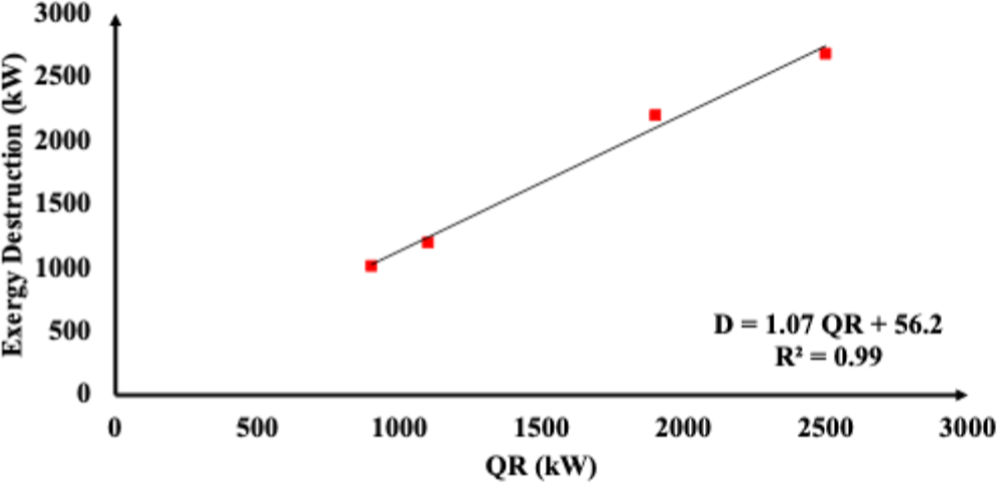
Exergy destruction as a function of reboiler heat duty.

Figures 7 and 8 illustrate the graphs for the de-ethanizer column (T-02) and debutanizer column (T-03), respectively. These figures show the exergy destruction, heat of the reboiler and condenser, and mass flow rates of the top and bottom products of the column. Column T-02 presents only 6% exergy destruction compared with column T-01 for all process diagram arrangements. Exergy destruction of column T-02 is related to the ethane flow rate, which results in an exergy destruction value of approximately 280 kW/(kg s^–1^) of ethane. Consequently, lower top product flow rates (*C*_2_) require less heat removal in the condenser, leading to lower exergy destruction. Similar observations can be found in column T-03. The absolute values of exergy destruction for T-03 are 61% higher than those of column T-01 due to higher flow rates of the top product (LPG) compared to the bottom product (*C*_5_^+^) in column T-02. The exergy destruction of T-03 is associated with the LPG separation flow rate, which is approximately 138 kW/(kg s^–1^) of LPG.

**Figure 7:**
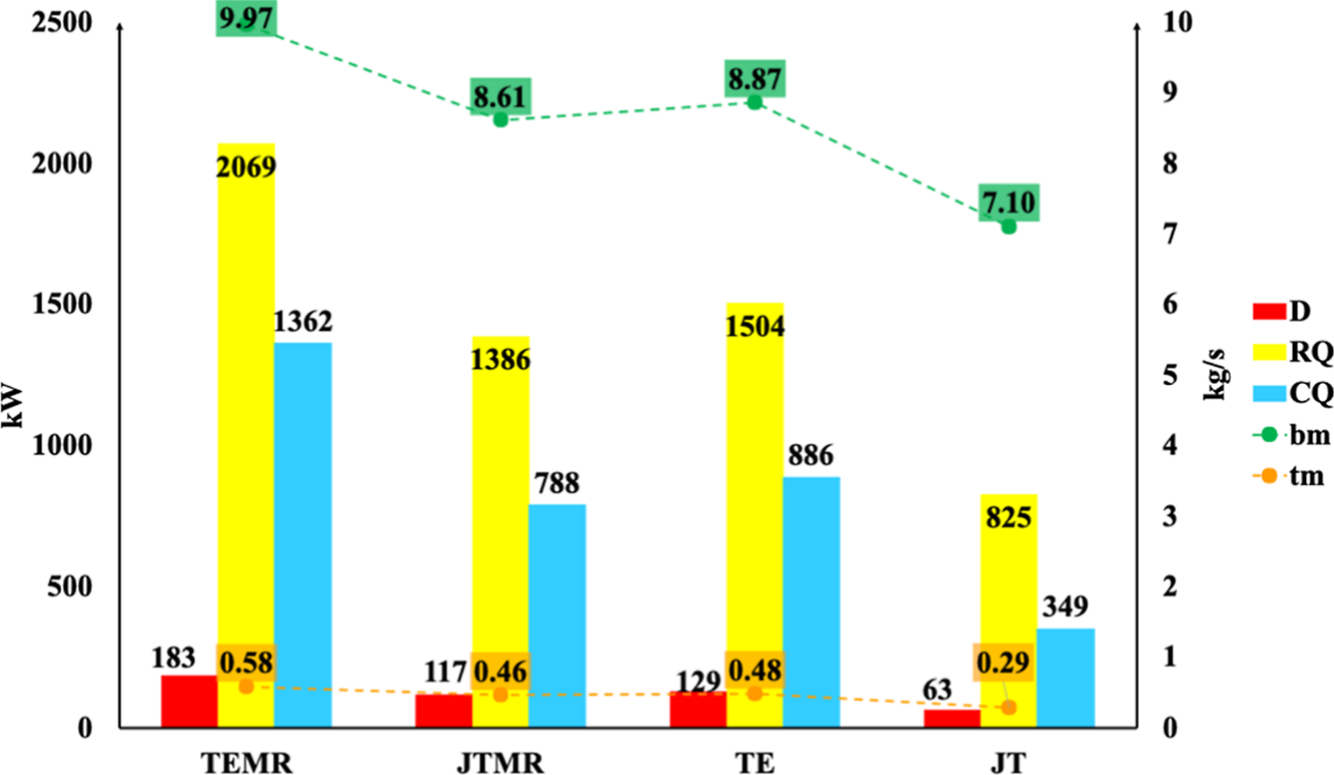
Exergy analysis for the T-02 de-ethanizer column. Exergy destruction (*D*), reboiler heat (QR), condenser heat (QC), and top (mt) and bottom (bm) product mass flow rates.

**Figure 8:**
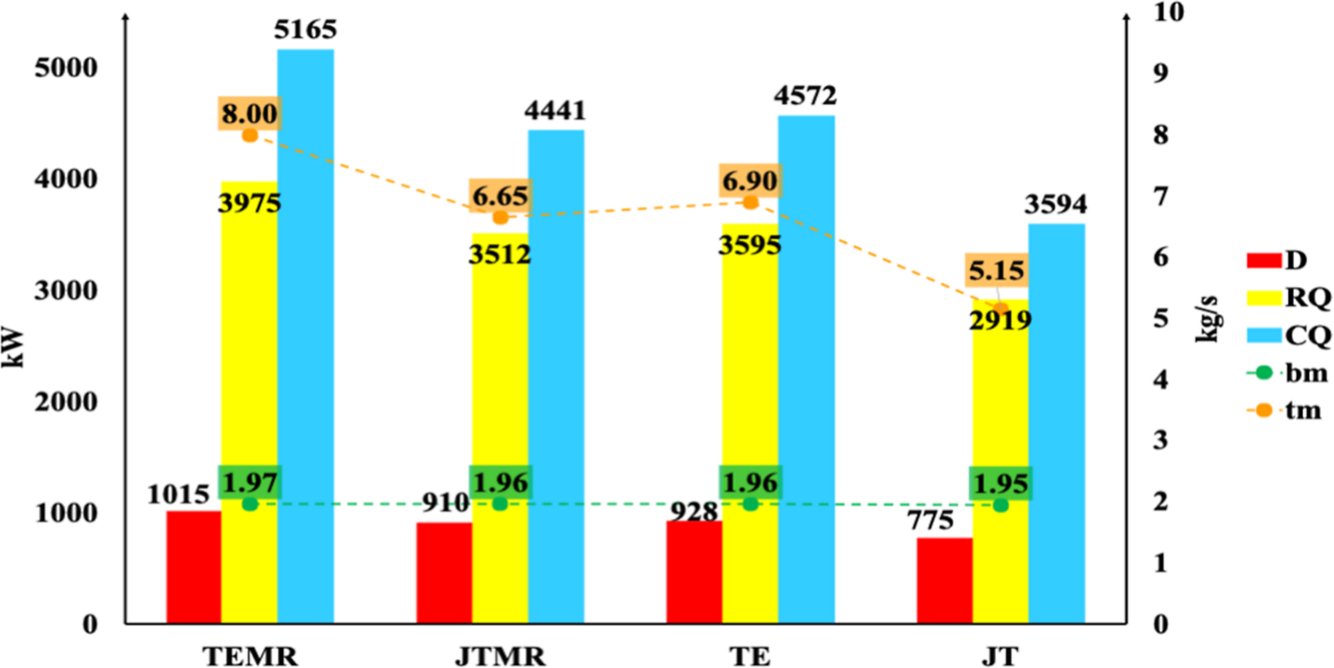
Exergy analysis for the T-03 debutanizer column. Exergy destruction (*D*), reboiler heat (QR), condenser heat (QC), and top (mt) and bottom (bm) product mass flow rates.

The exergy destruction of column T-01 is influenced by the tower inlet temperature, which is determined by the expansion process aided by mechanical refrigeration systems. As the TEMR system achieves better cooling of the gas feed stream, it results in the highest exergy destruction. However, for columns T-02 and T-03, exergy destruction only depends on the mass flow rate at the column top, regardless of the process arrangement.

#### 3.1.1 Exergy Efficiency of Equipment

Table 14 presents the exergy efficiencies achieved by all the equipment with their respective arrangements. Plate heat exchangers (P-01 to P-06) and chillers (P-07 and P-08) demonstrate high efficiency (99%).

**Table 14:** Exergy Efficiency of Equipment

	ε (%)			
equipment	TE	JT	TEMR	JTMR
P-01	99.66	99.66	99.63	99.63
P-03	99.84	99.85	99.62	99.71
P-05	99.04	99.40	99.22	99.45
P-06	99.38	99.50	99.56	99.65
JT-01		14.41		20.00
TE-01	69.77		67.03	
T-01	88.53	92.55	81.97	88.96
T-02	88.43	91.60	86.54	88.89
T-03	50.42	46.73	52.29	49.79
C-01	77.03		76.43	
C-02	81.73	81.42	81.76	81.52
AC-01	91.75	92.00	91.73	91.95
P-08	99.76	99.74	98.03	99.75
P-07	97.95	98.05	99.76	97.96

This indicates that all arrangements effectively integrate thermal energy between the product and feed gas streams within the heat exchangers. In the expansion, the Turbo-Expander (TE) surpasses the Joule–Thompson (JT) valve in efficiency. The exergy efficiency of the turbo-expander is 69% in TE and 67.03% in TEMR; however, the Joule–Thompson valve shows an efficiency of only 14% in the JT configuration and 20% in the JTMR, and the efficiencies of compressors C-01 and C-02 are comparable. For TE and TEMR, compressor C-02 has an average efficiency of 81%, while the additional stage compressor C-01 reaches 76%. Although both compressors have an isentropic efficiency of 75%, C-02 is considered more efficient because it generates a higher pressure in the gas stream than does C-01. For compressors, the greater the pressure changes, the greater the energy efficiency. In general, the air coolers achieve an efficiency of approximately 92% across all process arrangements. For column T-01, JT is the most efficient arrangement, while TEMR is the least efficient. In column T-02, the arrangement with the highest efficiency is JT (92%), whereas TEMR has the lowest efficiency (82%). These efficiencies correspond to the lower top product flow rates (*C*_2_) achieved by these arrangements. Column T-03 is identified as the least efficient, indicating that as the top product flow rate (LPG) increases, the efficiency of the column decreases.

Figure 9 illustrates the exergy efficiency (ε) of the TEMR and JTMR arrangements considering both cooling stages (P-02 and P-04). For both arrangements, the first stage has the lowest efficiency. This is primarily due to the cooling in this stage (P-02) being connected to the P-01 heat exchanger, which interacts with the sales gas stream and the feed gas inlet. In contrast, the P-04-s stage is more efficient because the feed gas passing through the P-05 heat exchanger is cooled by a colder sales gas stream.

**Figure 9:**
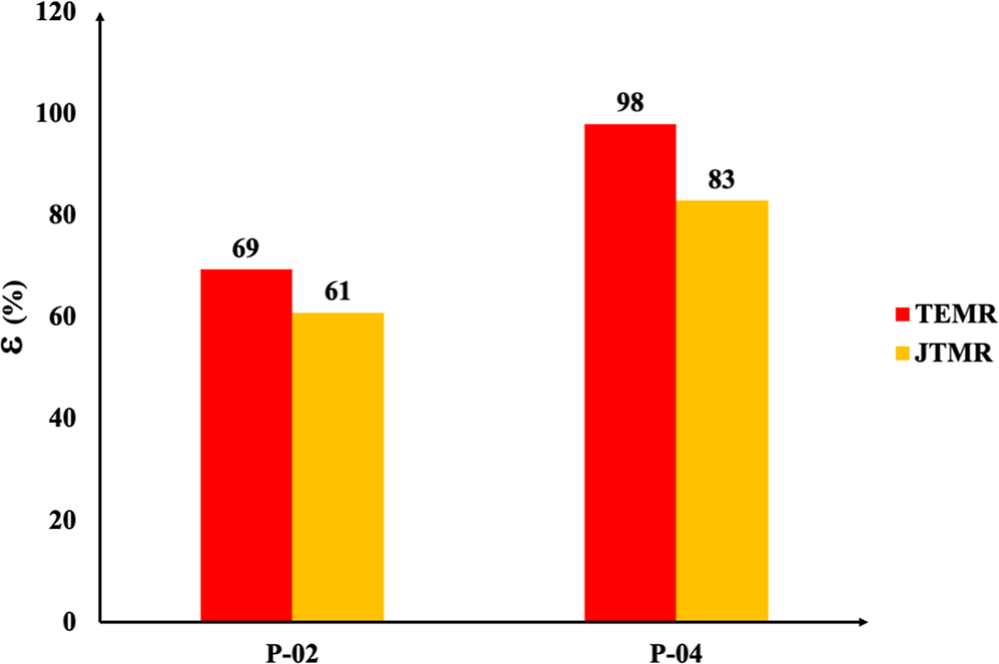
Exergy efficiency of mechanical refrigeration.

#### 3.1.2 Distribution of Exergy Destruction by Components

In relation to the equipment, Figure 10 illustrates the distribution of the exergy destruction for all arrangements. The TEMR and TE arrangements have higher exergy destruction in the columns, whereas the JTMR and JT occur during the expansion. The TE-01 Turbo-Expander cools the gas stream more effectively than the valve, resulting in lower temperatures at the inlet of the T-01 column. This, in turn, increases the heat rate requirement in the reboiler. Compared to the TE, the expansion valve used in JT and JTMR is less efficient than the Turbo-Expander as it operates through a completely irreversible process. The overall exergy destruction for compressors and air coolers is around 18%.

**Figure 10:**
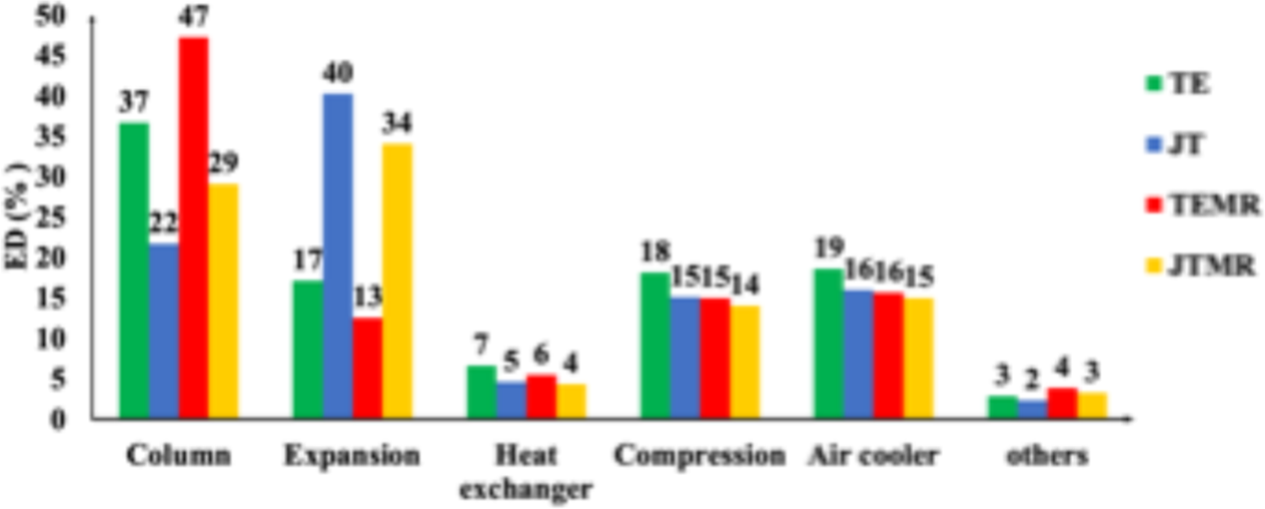
Distribution of exergy destruction by components in the arrangements.

Furthermore, since the columns, expansion, compression, and air coolers exhibit the highest levels of exergy destruction, it is essential to prioritize the reduction of exergy destruction in such equipment. Comparing these results to the work of Shamsi et al.,^24^ for instance, column T-01 can be optimized by utilizing heat from other streams or residual heat to lower its inlet temperature. This change reduces the energy consumption of the reboiler. In one of the configurations studied by Shamsi et al.,^24^ the two-column streams were employed, which returned at a higher temperature after passing through a heat exchanger with the feed gas. This approach reduces exergy destruction and minimizes heat waste from the reboiler. For compressors (C-01 and C-02), optimizing the operating pressure differential using multistage compressors is recommended.

#### 3.1.3 Global Exergy Efficiency

Table 15 summarizes the exergy efficiency for each process arrangement, highlighting that the TE arrangement is the most efficient. This superiority stems from two key factors. First, the TE arrangement utilizes the Turbo-Expander to generate shaft work, which is integrated into the process, making it more efficient than expansion systems using the Joule–Thomson valve (JT and JTMR). Second, the higher inlet temperature of column T-01 in the TE arrangement compared to that of the TEMR configuration further enhances its efficiency. These combined contributions establish the TE arrangement as the most exergy-efficient option. To further improve the overall efficiency of expansion systems, efforts should focus on either enhancing the performance of the JT-valve or optimizing the operating conditions of the columns.

**Table 15:** Global Exergy Efficiency

process	ε (%)
TEMR	66.74
JTMR	64.54
TE	70.84
JT	65.80

### 3.2 Exergoeconomic Analysis

Table 16 presents the destruction cost rate (CD), relative cost of fuel and product (*r*), and exergoeconomic factor (*f*) for four system arrangements. Comparing the *f* factor between the TE and JT arrangements reveals that TE-01 has a higher *f* value, indicating a need to reduce equipment costs to minimize overall system costs. Conversely, JT-01 has a lower *f* factor, suggesting a focus on efficiency improvement. Regarding the relative cost (*r*), TE-01 exhibits higher values than JT-01, highlighting the need to lower product costs, particularly for energy generation. TEMR shows similar trends to those of TE for TE-01. In contrast to JT, the JTMR has a lower *f* factor and a higher destruction cost rate, signifying lower efficiency for the JT-01 usage when compared with the JT-01.

**Table 16:** Results of Exergoeconomic Analyses for Each Arrangement Studied

	CD (US $/h)				*r* (%)				*f* (%)			
	TE	JT	TEMR	JTMR	TE	JT	TEMR	JTMR	TE	JT	TEMR	JTMR
P-01	132	176	129	137	0	0	0	0	50	45	50	50
P-03	132	145	129	137	0	0	0	0	50	50	50	50
P-06	132	145	129	137	0	0	0	0	50	50	50	50
P-05	132	145	129	137	0	0	0	0	50	50	50	50
V-01	31	35	179	130	5	5	3	3	76	76	35	44
TE-01	110		88		215		209		86		84	
JT-01		517.6		1120		96		12		26		10
T-01	303	218	2013	786	134	123	53	9	90	93	58	79
T-02	27	25	120	54	1028	921	284	515	99	99	94	98
T-03	1059	1296	1229	1201	251	117148	214	248	61	58	57	59
C-01	67		45		1516		1744		98		98	
C-02	175	233	189	228	916	912	869	865	98	97	97	97
AC-01	1459	1382	2113	1662	98	12	11	11	25	28	18	23
B-01	0	0	0	0	1514	14318	1324	1612	98	99	98	98
P-07	76	83	74	78	0	0	0	0	50	50	50	50
P-08	76	83	74	78	0	0	0	0	50	50	50	50
P-02			1	1			68	92			100	100
P-04			1	4			78	46			100	100

For T-01 and T-02 columns, the high f factors for both the TE and JT arrangements indicate reducing equipment costs. Despite T-02 elevated relative cost, which points to the need for optimization, Figure 8 suggests that this is linked to low ethane production. This explains the low column destruction cost and high relative cost. For T-03, the low *f* factor indicates a lower investment cost relative to its destruction cost rate, suggesting the need for improved exergy efficiency. Strategies could include integrating exergy with heat recovery between process streams and the column feed or optimizing the energy use in the condenser and reboiler.

For TEMR, T-01 displays low *f* factors and high destruction costs, indicating the need to improve the column exergy performance. However, columns T-02 and T-03 in TEMR demonstrate an exergoeconomic performance comparable to that of TE and JT.

Across all arrangements, compressors C-01 and C-02 have high relative cost factors, emphasizing the need to reduce and optimize equipment costs. This can be achieved by purchasing lower-cost equipment and improving the isentropic efficiency to maintain pressure variations with reduced energy consumption.

For heat exchangers, the cost factor is 50%, indicating that the investment cost equals the exergy destruction cost. Reducing the equipment costs could also lower exergy destruction costs.

The AC-01 air cooler has a low-cost factor, however, high destruction costs. The efficiency could be further improved by recovering heat from the sales gas stream, even though the cooler is already highly efficient (92%).

As noted, the exergoeconomic factor (%f) remains consistently around 80% for TE and TEMR arrangements. However, TEMR attains the highest cost rate ($5687/h), primarily due to the T-01-purchased costs. In contrast to TEMR, the TE has the lowest exergy destruction cost ($3948/h), indicating TE as the most favorable arrangement from an exergoeconomic standpoint.

### 3.3 Model Validation and NGL Process Comparisons

With respect to model validation, Aspen HYSYS was selected by Junior et al.^26^ for process simulation due to its widespread application and validation in cryogenic NGL recovery modeling, as adopted in recent works such as Islam et al.^25^ As a validation strategy, key performance indicators such as exergy efficiency and NGL recovery rates from the current simulations were compared with the basic literature for model construction. The exergy efficiency for the GSP-TE arrangement in this study (71%) closely aligns with values reported by Getu et al.^10^ (70.5–73.5%) and Kherbeck and Chebbi^28^ (69–72%). Similarly, the NGL recovery rates achieved (up to 86%) are consistent with the ranges of 85–90%,^25^ 84–88%,^43^ and 83–89%^30^ for similar process configurations. This cross-validation reinforces the reliability, accuracy, and applicability of the chosen Aspen HYSYS modeling approach for the Brazilian market context.

In addition, Table 17 shows the performance of different processes for NGL recovery based on other indicators, such as ethane, energy efficiency, and specific energy consumption.

**Table 17:** NGL Process Comparisons Considering Ethane, Energy Efficiency, and Specific Energy Consumption

process	ethane recovery (%)	overall exergy efficiency (%)	specific energy consumption (kW h/kg NGL)	reference
present research	79.3	71.0	0.27	
RSV	81.2	73.5	0.31	^44^
IPSI-1	84.0	75.2	0.35	^8^
GSP	74.6	67.2	0.26	^25^
CRR	78.5	70.1	0.28	^10^

Comparisons enhance the credibility of the model against simulation results and industrial benchmarks. Our current research distinguishes itself from other GSP simulations by Islam et al.,^25^ emphasizing ethane recovery and overall exergy efficiency while keeping the specific energy recovery within the same order of magnitude. In contrast, IPSI-1 achieves the highest ethane recovery and exergy efficiency but has the most significant specific energy consumption.

Regarding the exergy and exergoeconomic perspectives, a comparative analysis with existing literature was conducted, considering exergy efficiency, exergy destruction cost, and NGL recovery rates. The present research evaluated four GSP-based configurations, with the Turbo-Expander (TE) achieving the highest exergy efficiency (71%) and NGL recovery (up to 86%). In contrast, the TEMR configuration exhibited the highest exergy destruction cost (5687 US $/h).

Compared to Islam et al.,^25^ which reports exergy efficiencies of up to 67% and NGL recoveries of 93% for IPSI-1 without exergoeconomic data, this work integrates thermodynamic and economic performance indicators. Shoghl et al.^43^ presented high valve-specific exergy efficiencies (91.6–98.9%) for Joule–Thomson stages; however, the results lacked global exergoeconomic metrics. Similarly, Hu et al.^30^ reported comparable exergy efficiencies (∼70% for HPA configurations) without quantifying exergy destruction costs. Jovijari et al.^45^ conducted an analysis of a real NGL plant, detailing the exergy destruction attributed to each piece of equipment. However, it did not include a monetary conversion for exergoeconomic valuation. Getu et al.^10^ performed a techno-economic assessment of several recovery schemes but did not report exergy or exergoeconomic performance. Ghorbani et al.^37^ evaluated integrated NGL-LNG processes, focusing on exergy distribution, without a monetized exergoeconomic analysis.

Based on process comparisons, the results obtained in this research align with the values reported in the literature for similar processes. The exergy efficiencies obtained for the TE configuration (71%) are consistent with values reported for HPA (70%) by Hu et al.^30^ and IPSI-1 (67%) by Islam et al.,^25^ validating the thermodynamic consistency of the simulation results. Furthermore, the NGL recovery rates (up to 86%) achieved in this study align with the 85–93% recovery levels reported in Hu et al.^30^ and Islam et al.^25^

Regarding the exergy destruction values for individual equipment, particularly the cost of destruction in TEMR scenarios shows expected trends associated with exergoeconomic sensitivities identified in Jovijari et al.^45^ and Islam et al.^25^ Moreover, it is important to highlight that this research is the only one to simultaneously report global exergy efficiencies, exergy destruction costs in US $/h, and NGL recovery rates for multiple GSP configurations contextualized to the Brazilian market. This integrated exergoeconomic and process optimization assessment offers a valuable reference for improving the operational performance and economic feasibility in emerging gas markets.

## 4 Conclusions

The GSP, commonly used in Brazil, offers flexibility in meeting market demands for various hydrocarbon products including LPG, natural gasoline, ethane, and methane for thermoelectric plants. However, challenges, such as high energy consumption and inefficiencies within the process, require further optimization. Therefore, this research novelty highlights exergy and exergoeconomic analyses for optimal profitability throughout their operational lifespan. In conclusion, the turbo-expansion (TE) arrangement achieves the highest exergy efficiency (71%) and the lowest exergy destruction cost (111 US $/h), making it a solution for natural gas process design. The exergoeconomic analysis revealed that reducing operating and maintenance costs, especially for equipment with high exergoeconomic factors, is crucial for ensuring the economic viability of these arrangements. Equipment such as the Joule–Thompson valve and air coolers, which have low exergoeconomic factors (JT-01, 26%; AC-01, 28%), requires enhancements in exergy efficiency to minimize exergy destruction and related costs. Moreover, such an analysis contributes to efficient and sustainable process design, supporting the advancement of the energy transition and enhancing the competitiveness of the global natural gas industry.

## Supplementary Material



## Notes

Detailed data on material and energy streams, capital and operating costs, and exergy costs for the gas-subcooled process (GSP) flow diagram (PDF)
