# Supplementary material for: Exergy and Exergoeconomic Analysis of the Gas Subcooled Process for the Brazilian Market: An Approach to Enhance Natural Gas Liquids Recovery
Source: ACS Omega. 2025 Jun 24;10(26):28092–111. doi: 10.1021/acsomega.5c02484 (PMC12242666; doi:10.1021/acsomega.5c02484)
Supplement: Supplementary file 1 [file ao5c02484_si_001.pdf]

Supporting information file

Exergy and exergoeconomic analysis of gas subcooled process for the Brazilian market: an approach to enhance natural gas liquids recovery

ISIDRO ALEJANDRO ARGUETA FLORES[1]; ANA PAULA MENEGUELO[1]; CINTIA MARANGONI[2]; YURI NASCIMENTO NARIYOSHI[3]; MARCELO SILVEIRA BACELOS[1*]

1- Universidade Federal do Espírito Santo, Departamento de Engenharias e Tecnologias, Programa de Pós-graduação em Energia, Rod. BR 101 Norte, Km. 60, 29932-540, São Mateus–ES, Brasil,

2- Universidade Federal de Santa Catarina, Departamento de Engenharia Química e Alimentos, Programa de Pós-graduação em Engenharia Química e Alimentos, Campus Universitário, Trindade, 88040-900, Florianópolis – SC, Brasil

3- Universidade Federal do Espírito Santo, Departamento de Engenharia Ambiental, Campus Universitário, Av. Fernando Ferrari 514, 29075-910, Vitória–ES, Brasil

***Corresponding author:** Marcelo Silveira Bacelos; e-mail address: marcelo.bacelos@ufes.br

This supporting information file provides detailed data on material and energy streams, capital and operating costs, and exergy costs for the Gas-Subcooled process (GSP) flow diagram.

Contents

Table S1- Exergy of the stream for each arrangement

Table S2 – Exergy of energy stream for each arrangement

Table S3- Capital investment and operation and maintenance cost for each equipment.

Table S4- Purchase cost of the process components

Table S5- Exergy cost and unit cost of material streams for each arrangement

Table S1- Exergy of the stream for each arrangement

Stream	Exergy (kW)			
	TE	JT	TEMR	JTMR
1	16878.61	16877.90	16885.38	16885.12
2	16872.70	16869.91	16903.94	16898.18
3	16968.18	16951.66	17010.43	16973.24
4	1086.05	991.00	17283.38	17044.77
5	15670.95	15760.34	17551.27	17161.41
6	966.82	880.96	3922.93	2127.98
7	3714.02	3735.20	3564.38	14738.42
8	11956.95	12025.16	13290.65	1916.15
9	9614.20	9260.58	3149.88	3493.00
10	4132.16	3986.78	3700.94	11245.44
11	3683.83	3409.42	3497.07	3864.40
12	12127.97	12152.36	10140.74	8794.88
13	11557.17	11805.38	8527.53	3445.11
14	11284.47	11580.49	12355.27	12136.98
15	11245.85	11545.29	11683.30	11679.49
16	11158.10	11456.93	11288.97	11460.95
17	11158.10	11456.93	11076.18	11302.59
18	0.00	0.00	10974.80	11200.93
19	12417.22	17133.97	10974.80	11200.93
20	16776.80	593.18	0.00	0.00
21	833.16	599.61	11801.26	16797.73
22	841.38	586.14	16530.83	798.13
23	792.12	576.61	979.62	806.09
24	779.92	601.80	988.94	760.59
25	821.03	110.70	924.76	748.79
26	111.51		910.93	786.72
27			967.17	111.53
28			111.80	
Ethane (C2)	120.90	71.43	145.01	115.05
C5+	52.10	72.13	35.37	56.02
LGP	650.11	482.71	758.88	624.16
SG	15392.24	15762.44	15163.94	15445.58
NGL	823.03	585.40	968.05	788.34
Gás	16886.35	16886.35	16886.35	16886.35

Table S2 – Exergy of energy stream for each arrangement

Stream	Exergy (kW)			
	TE	JT	TEMR	JTMR
B-01	13.09	10.08	14.93	12.66
C-01	1634.63		1081.36	
C-02	5334.15	6972.92	5784.50	6865.71
E T-01	364.61	211.48	665.80	372.62
E T-02	270.95	148.59	372.61	249.64
E T-03	1050.04	852.48	1160.88	1025.82
Q P-02			26.78	21.48
Q P-04			278.69	86.37
Q T-02	70.65	27.81	108.45	62.82
Q T-03	181.85	86.13	242.08	166.75
TE-01	1634.63		1081.36	

Table S3- Capital investment and operation and maintenance cost for each equipment.

Equipment	ZCL (US$/h)				ZOML(US$/h)				Z (US$/h)			
	**TE**	**JT**	**TEMR**	**JTRM**	**TE**	**JT**	**TEMR**	**JTRM**	**TE**	**JT**	**TEMR**	**JTRM**
P-01	107.83	116.39	106.19	110.88	24.91	29.39	23.58	26.54	132.74	145.77	129.77	137.41
P-03	107.83	116.39	106.19	110.88	24.91	29.39	23.58	26.54	132.74	145.77	129.77	137.41
P-06	107.83	116.39	106.19	110.88	24.91	29.39	23.58	26.54	132.74	145.77	129.77	137.41
P-05	107.83	116.39	106.19	110.88	24.91	29.39	23.58	26.54	132.74	145.77	129.77	137.41
V-01	82.03	88.54	80.78	84.35	18.95	22.36	17.94	20.19	100.98	110.90	98.72	104.54
V-02	82.03	88.54	80.78	84.35	18.95	22.36	17.94	20.19	100.98	110.90	98.72	104.54
TE-01	536.66		378.19		124.00		83.96		660.67		462.15	
JT-01		144.82		98.72		36.56		23.63		181.38		122.35
T-01	2302.71	2485.54	2267.78	2367.86	532.07	627.55	503.49	566.69	2834.79	3113.09	2771.27	2934.55
T-02	1705.39	1840.79	1679.52	1753.65	394.06	464.77	372.88	419.69	2099.45	2305.56	2052.41	2173.34
T-03	1337.27	1443.44	1316.98	1375.11	309.00	364.44	292.39	329.10	1646.27	1807.88	1609.38	1704.20
C-01	2735.24		2084.96		632.02		462.90		3367.26		2547.86	
C-02	5694.51	7257.27	5897.15	6847.59	1315.80	1832.32	1309.27	1638.81	7010.31	9089.60	7206.42	8486.40
AC-01	391.16	422.22	385.23	402.23	90.38	106.60	85.53	96.26	481.55	528.82	470.76	498.50
B-01	21.98	22.91	22.06	22.50	5.08	5.79	4.90	5.39	27.06	28.70	26.96	27.89
P-07	61.73	66.64	60.80	63.48	14.26	16.82	13.50	15.19	76.00	83.46	74.30	78.67
P-08	61.73	66.64	60.80	63.48	14.26	16.82	13.50	15.19	76.00	83.46	74.30	78.67
P-02			1859.21	1748.42			412.78	418.44			2271.99	2166.87
P-04			3547.71	1985.79			787.66	475.25			4335.36	2461.04

Table S4- Purchase cost of the process components.

Equipment	PEC (USk)			
	TE	JT	TEMR	JTRM
P-01	44.06	44.06	44.06	44.06
P-03	44.06	44.06	44.06	44.06
P-06	44.06	44.06	44.06	44.06
P-05	44.06	44.06	44.06	44.06
V-01	33.52	33.52	33.52	33.52
V-02	33.52	33.52	33.52	33.52
TE-01	219.29		156.91	
JT-01		54.82		39.23
T-01	940.92	940.92	940.92	940.92
T-02	696.85	696.85	696.85	696.85
T-03	546.43	546.43	546.43	546.43
C-01	1117.66		865.07	
C-02	2326.86	2747.30	2446.78	2721.04
AC-01	159.84	159.84	159.84	159.84
B-01	8.98	8.67	9.15	8.94
P-07	25.23	25.23	25.23	25.23
P-08	25.23	25.23	25.23	25.23
P-02			771.40	694.77
P-04			1471.98	789.09

Table S5- Exergy cost and unit cost of material streams for each arrangement

Stream	C(US$/h)				c(US$/Gj)			
	TE	JT	TEMR	JTMR	TE	JT	TEMR	JTMR
1	2575.80	3081.63	3295.37	3098.67	42.39	50.72	54.21	50.98
2	2593.42	3108.62	5523.71	5264.47	42.70	51.19	90.77	86.54
3	2521.91	3025.36	5393.69	5308.65	41.28	49.57	88.08	86.88
4	169.99	185.54	9611.10	7765.37	43.48	52.01	154.47	126.55
5	2452.90	2950.72	9336.18	7608.06	43.48	52.01	147.76	123.15
6	169.99	185.54	2150.20	973.07	48.84	58.50	152.25	127.02
7	581.33	699.32	2150.20	6739.52	43.48	52.01	167.57	127.02
8	1871.56	2251.40	7284.71	973.07	43.48	52.01	152.25	141.06
9	2238.00	2432.78	2212.68	1597.26	64.66	72.97	195.13	127.02
10	217.75	314.96	1621.26	5142.26	14.64	21.94	121.69	127.02
11	217.75	314.96	1621.26	1004.49	16.42	25.66	128.78	72.20
12	5130.58	5778.58	7123.51	5264.61	117.51	132.09	195.13	166.28
13	5626.91	6308.70	7391.02	1004.49	135.24	148.44	240.76	80.99
14	5831.17	6537.74	12952.21	9573.52	143.54	156.82	291.20	219.11
15	5946.28	6656.52	13673.39	10303.71	146.88	160.15	325.09	245.06
16	6068.26	6817.70	14078.07	10598.43	151.07	165.30	346.41	256.87
17	6047.26	6928.59	14337.85	10691.65	150.54	167.99	359.58	262.76
18			14466.39	10827.43			366.15	268.52
19	9708.76	17273.31	14565.11	10931.96	217.19	280.04	368.65	271.11
20	17679.21	480.43			292.72	224.98		
21	377.59	307.57	17307.62	20654.19	125.89	142.49	407.39	341.55
22	220.47	2334.63	25555.25	652.00	72.79	1106.41	429.42	226.92
23	2021.40	2590.95	1044.47	488.33	708.86	1248.17	296.17	168.28
24	2254.52	2433.89	890.79	2320.08	802.98	1123.43	250.21	847.33
25	2083.82	798.44	2555.65	2562.41	705.02	2003.54	767.66	950.58
26	552.47		2783.62	2392.65	1376.27		848.83	844.81
27			2576.59	627.58			740.01	1563.09
28			543.38				1350.13	
Ethane (C2)	18160.76	17802.14	26026.00	21152.68	327.74	313.72	476.75	380.42
C5+	348.17	278.36	1014.82	621.83	117.51	132.09	291.20	219.11
LGP	308.53	284.53	400.76	350.94	708.86	1106.41	767.66	847.33
SG	3221.03	3481.65	3688.53	3512.22	1376.27	2003.54	1350.13	1563.09
NGL	799.17	1038.96	824.71	876.02	4261.28	4001.33	6476.15	4343.66
Gás	2565.04	3097.04	3294.14	3097.04	42.19	50.95	54.19	50.95
